# Binding of Ca^2+^-independent C2 domains to lipid membranes: A multi-scale molecular dynamics study

**DOI:** 10.1016/j.str.2021.05.011

**Published:** 2021-10-07

**Authors:** Andreas Haahr Larsen, Mark S.P. Sansom

**Affiliations:** 1 Department of Biochemistry, University of Oxford, South Parks Road, Oxford OX1 3QU, UK

**Keywords:** C2 domain, PIP_2_, membrane, MD simulation, coarse-grained, lipid

## Abstract

C2 domains facilitate protein interactions with lipid bilayers in either a Ca^2+^-dependent or -independent manner. We used molecular dynamics (MD) simulations to explore six Ca^2+^-independent C2 domains, from KIBRA, PI3KC2α, RIM2, PTEN, SHIP2, and Smurf2. In coarse-grained MD simulations these C2 domains formed transient interactions with zwitterionic bilayers, compared with longer-lived interactions with anionic bilayers containing phosphatidylinositol bisphosphate (PIP_2_). Type I C2 domains bound non-canonically via the front, back, or side of the β sandwich, whereas type II C2 domains bound canonically, via the top loops. C2 domains interacted strongly with membranes containing PIP_2_, causing bound anionic lipids to cluster around the protein. Binding modes were refined via atomistic simulations. For PTEN and SHIP2, CG simulations of their phosphatase plus C2 domains with PIP_2_-containing bilayers were also performed, and the roles of the two domains in membrane localization compared. These studies establish a simulation protocol for membrane-recognition proteins.

## Highlights

• Binding of Ca^2+^-independent C2 domains to membranes was explored by MD simulation
• C2 domains from KIBRA, PI3KC2α, RIM2, PTEN, SHIP2, and Smurf2 were compared
• C2 domains formed longer-lived interactions with lipid bilayers containing PIP_2_
• For PTEN and SHIP2, simulations of their phosphatase plus C2 domains were performed

## Graphical abstract

**Figure:**
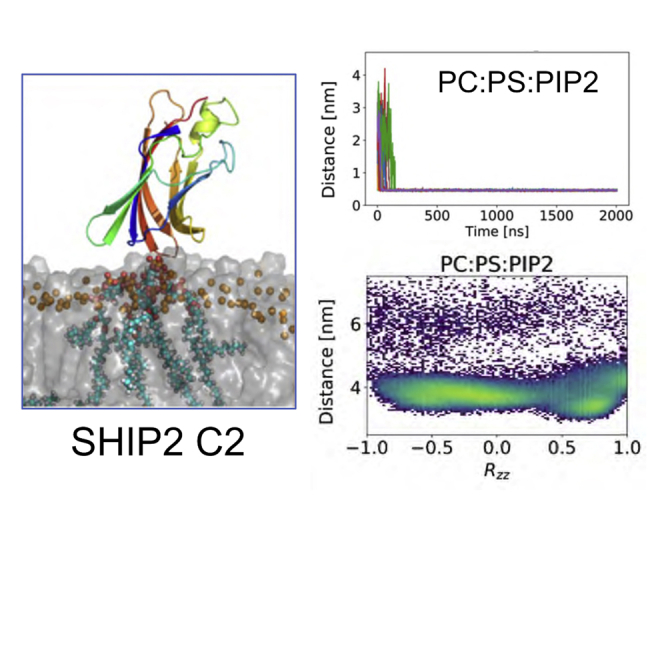

## Teaser

Larsen and Sansom analyzed MD simulations of the interactions of six different Ca^2+^-independent C2 domains with lipid bilayers. C2 domains interacted strongly with membranes containing PIP_2_. For PTEN and SHIP2, simulations of their phosphatase plus C2 domains allowed the roles of the two domains in membrane localization to be compared.

## Introduction

Lipid-specific membrane recognition plays a key role in the biology of eukaryotic cells. A number of families of recognition domains exist. Of especial importance are domains that recognize phosphoinositide (PI) lipids (Kutateladze, 2010; Stahelin et al., 2014), with C2 and PH domains being the most abundant families (Katan and Allen, 1999). C2 domains can enable both protein-lipid and protein-protein interactions (Corbalan-Garcia and Gómez-Fernández, 2014; Nalefski and Falke, 1996; Zhang and Aravind, 2010) and are key players in a number of cellular signaling processes involving, e.g., ubiquitination (Wiesner et al., 2007) or (de)phosphorylation (Chen et al., 2018; Gericke et al., 2013). C2 domains bind membranes and/or other proteins and thereby bring, e.g., adjacent catalytic domains into contact with their interaction partners. One intensively studied example of a C2-domain-containing protein is the phosphatase and tensin homolog (PTEN), which consists of a C2 domain and a phosphatase domain (Lee et al., 1999; Maehama and Dixon, 1999; Zhao et al., 2004). In PTEN, C2 provides a lipid-mediated anchor to the membrane, so the phosphatase domain can dephosphorylate phosphatidylinositol triphosphate (PIP_3_) to phosphatidylinositol 3,4-diphosphate (PI(3,4)P_2_). This dephosphorylation downregulates the Akt pathway, and PTEN is thus a tumor suppressor.

**Figure 1:**
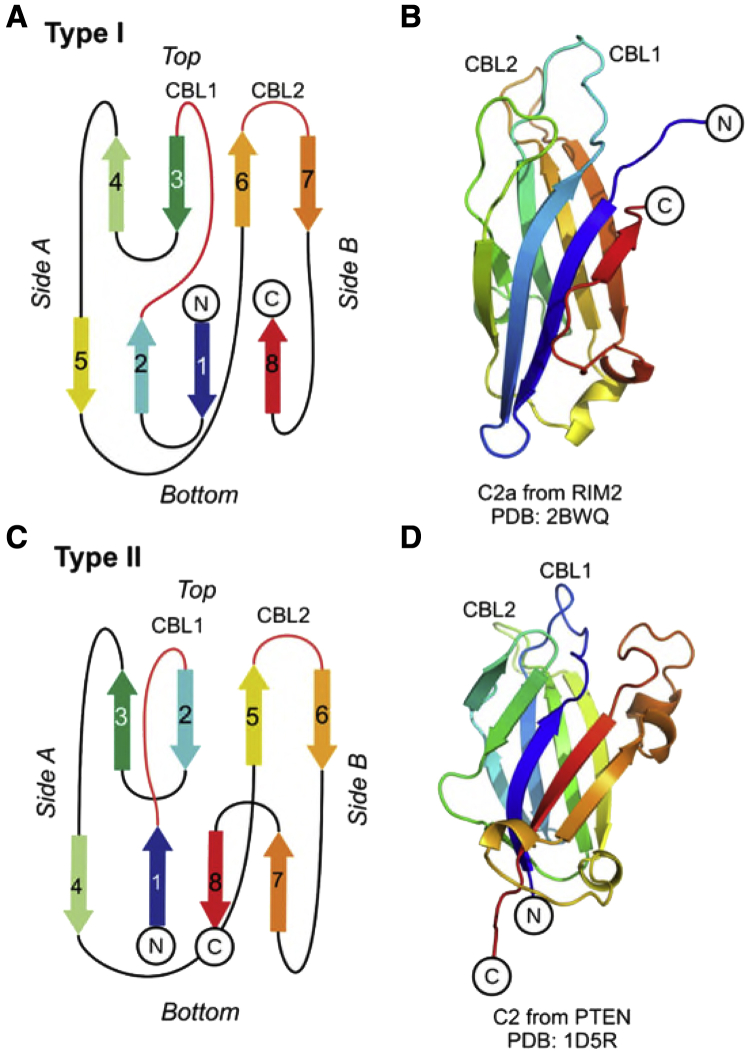
C2 structures and topologies (A and C) Schematic diagrams of the topologies of type I and type II C2 domains, with β strands numbered and connected with loops, colored using a rainbow spectrum, from blue to red. The β sheet containing the N and C termini is here denoted the “front” and the other β sheet the “back.” (B and D) Examples of C2 domains of each topology: the type I example is C2a from RIM2 (PDB: 2BWQ) and the type II example is C2 from PTEN (PDB: ID5R).

All C2 domains have a similar structure (Figure 1). They are composed of around 130 residues arranged as eight β antiparallel strands (β1–β8) in two β sheets, forming a β sandwich. C2 domains have two topologies, type I or type II, which differ by a circular permutation (Corbalan-Garcia and Gómez-Fernández, 2014; Nalefski and Falke, 1996). Calcium ions control the function of many C2-domains via Ca^2+^-mediated binding and unbinding to lipid membranes (Corbalan-Garcia and Gómez-Fernández, 2014). However, there is a group of Ca^2+^-independent C2 domains with either no or little Ca^2+^ dependency. These are the focus of the current study.

We examine how Ca^2+^-independent C2 domains interact with lipid membranes, especially those containing PI lipids. Molecular dynamics (MD) simulations have emerged as a powerful tool to examine the interactions of membrane proteins with lipids (Corradi et al., 2019; Hedger and Sansom, 2016; Manna et al., 2019) and have previously been applied to investigate the interactions of peripheral membrane proteins, especially PH domains, with PI-containing lipid bilayers (Kalli and Sansom, 2014; Lai et al., 2010, 2013; Naughton et al., 2016; Pant and Tajkhorshid, 2020; Yamamoto et al., 2016). There have been a number of MD studies of the interaction of Ca^2+^-dependent C2 domains with membranes, revealing how Ca^2+^ ions mediate interactions between the protein and anionic lipids such as phosphatidylserine (PS) and phosphatidylinositol phosphates (PIPs) (Alwarawrah and Wereszczynski, 2017; Banci et al., 2002; Chon et al., 2015; Jaud et al., 2007; Lai et al., 2010; Manna et al., 2008; Michaeli et al., 2017; Vermaas and Tajkhorshid, 2017). However, there have not been many simulation studies of the interactions of Ca^2+^-independent C2 domains with membranes (see, e.g., Alnaas et al., 2021; Scott et al., 2020) other than for PTEN (Shenoy et al., 2012a; Treece et al., 2020), despite the growing recognition of the importance of such interactions in a number of cellular processes (Stahelin et al., 2014). Here, we use coarse-grained molecular dynamics (CG-MD) (Ingólfsson et al., 2014; Marrink and Tieleman, 2013) to examine and compare the interactions of six different Ca^2+^-independent C2 domains with lipid bilayers: C2 domains from KIBRA (PDB: 6FJD), PI3KC2α (PDB: 6BU0), RIM2 (PDB: 2BWQ), PTEN (PDB: 1D5R), Smurf2 (PDB: 2JQZ), and SHIP2 (PDB: 5OKM). More complete structures are known for PTEN and SHIP2, so for these systems we also simulate the corresponding multi-domain protein and compare these with the simulations of isolated C2 domains. We explore both lipid specificity and the energetics of interaction. The most favorable interaction mode(s) for each C2 domain are refined by atomistic molecular dynamics (AT-MD) simulations. Based on ∼1 ms total of CG simulation data for six C2 domains and three membranes, we demonstrate that the binding orientations are related to topology, and we show that the C2 domains only remain bound to negatively charged membranes and can induce clustering of multiple phosphatidylinositol diphosphates. Our studies also provide a protocol for simulation studies of the specificity and energetics of lipid bilayer interactions of other families of membrane binding/recognition proteins.

## Results

### A protocol for determining and analyzing C2-domain-binding modes

**Figure 2:**
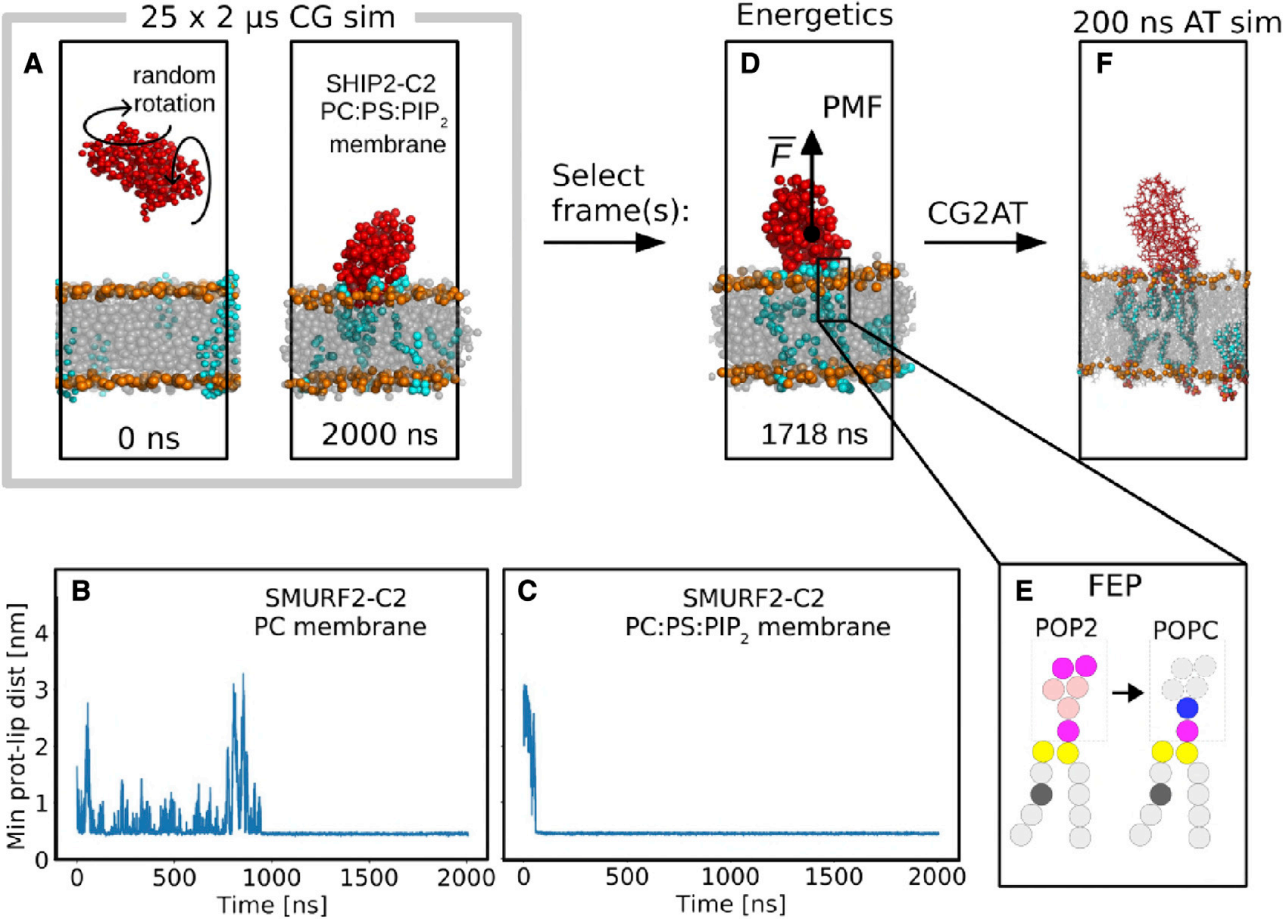
Simulation pipeline (A) 25 replica CG simulations (each of 2 μs duration) of C2 domain/membrane association were run for each system. First and last frames are shown for the C2 from SHIP2 with a PC:PS:PIP_2_ (80:15:5) membrane with PC and PS in gray, PIP_2_ in cyan, and beads representing phosphate groups in orange. (B and C) The minimum distance between the C2 domain and lipids of the bilayer for a single simulation (from an ensemble of 25 repeats) of the interaction of C2 from Smurf2 and either (B) a PC membrane or (C) a PC:PS:PIP_2_ (80:15:5) membrane. (D) Representative binding mode(s) were selected for each system as described in the main text. Potentials of mean force (PMF), i.e., free energy profiles, for the C2 domain/membrane interaction were calculated using umbrella sampling. (E) Free energy perturbation (FEP) calculations were performed, whereby PIP_2_ in the upper leaflet interacting with the bound C2 domain was converted to a PC and the free energy change evaluated. (F) Representative binding modes were converted from CG (force field MARTINI 2.2) to atomistic (force field CHARMM 36m with TIP3P waters) representation, and atomistic simulations were run for 200 ns.

To explore possible binding modes of C2 domains to lipid bilayers without bias from the initial simulation system configuration, we initiated CG simulations with the C2 domain positioned at sufficient distance that it could not “feel” the membrane, i.e., at a distance greater than the cutoff distances of the CG force field. For each simulation of an ensemble, the protein molecule was rotated through a random angle before the simulation was started (Figure 2). Each simulation was run for 2 μs, which proved long enough for the protein to encounter and interact with the bilayer, in some cases (dependent on the bilayer lipid composition) being able to dissociate and rebind. To ensure adequate sampling, based on previous experience with PH domains (Yamamoto et al., 2016) and other membrane proteins (e.g., phosphatidylinositol phosphate kinase PIP5K1A [Amos et al., 2019]), 25 repeats were run for each system. Thus, for six different C2 domains each with three different lipid membranes, a total of just under a millisecond of CG-MD simulations were performed. These were subsequently analyzed in terms of the binding orientation of the C2 domain relative to the membrane, the dependence on the membrane-lipid composition, and the energetics of the interaction.

### CG simulations of C2/membrane encounter

Six C2 domains were investigated, three type I and three type II (Table 1). The type I C2 domains were from KIBRA (PDB: 6FJD), PI3KC2α (PDB: 6BU0), and RIM2 (PDB: 2BWQ), and the type II C2 domains from PTEN (PDB: 1D5R), Smurf2 (PDB: 2JQZ), and SHIP2 (PDB: 5OKM). Here we focus on a comparative overview of their simulated interactions with lipid bilayers of varying composition. Three types of membranes were included in the study: zwitterionic (phosphatidylcholine [PC]), anionic (80% PC and 20% PS), and anionic membrane including a PI lipid (80% PC, 15% PS, and 5% phosphatidylinositol bisphosphate [PIP_2_]). We use the shorthand notations PC, PC:PS, and PC:PS:PIP_2_ to denote these membranes. PC:PS:PIP_2_ is a model membrane for the negatively charged inner leaflet of mammalian plasma human membrane, which is relevant as all the C2 domains under consideration bind to this membrane *in vivo*. The PIP_2_ content (5%) is somewhat higher than typical overall values of a plasma membrane (1%–2%) but is comparable with the concentration of PI lipids used for *in vitro* studies of C2 binding to a plasma membrane mimic (e.g., Stahelin et al., 2003). As our simulation box is rather small, we increased the PIP_2_ content to allow for the possibility of multiple PIP_2_ molecules clustering around a bound C2 domain.

**Table 1:** Summary of CG association simulations and PMFs

Domain	PDB ID	Topology	Binding orientation	PMF well depth^a^ (kJ/mol)		
				PC	PC:PS	PC:PS:PIP_2_
KIBRA-C2	6FJD	type I	side B	9.8 ± 1.0	26.0 ± 1.5	48.0 ± 1.4
PI3KC2α-C2b	6BUO	type I	front	15.0 ± 0.9	28.6 ± 1.3	64.1 ± 1.2
			back	22.7 ± 0.6	42.8 ± 0.7	115.2 ± 2.6
RIM2-C2a	2BWQ	type I	side A	32.5 ± 1.1	49.8 ± 0.9	120.4 ± 2.4
PTEN-C2	1D5R	type II	top	15.4 ± 2.7	27.0 ± 4.2	77.7 ± 4.3
SHIP2-C2	5OKM	type II	top	8.5 ± 1.1	10.4 ± 0.9	23.1 ± 1.0
Smurf2-C2	2JQZ	type II	top	19.9 ± 2.3	38.2 ± 1.0	81.5 ± 2.0

a As defined in Figure S4.

As can be seen from the example of the Smurf2-C2 domain (Figures 2B and 2C), a single simulation in the presence of either a PC or a PC:PS:PIP_2_ bilayer can be used to demonstrate the influence of the lipid bilayer composition on the protein-membrane interaction by monitoring the minimum distance between the protein and bilayer as a function of time. In this example, the C2 domain makes multiple encounters with the PC bilayer before finally binding to the surface after ∼1 μs (Figure 2B). In contrast, in the presence of the PI-containing bilayer, the initial encounter occurs within the first 0.1 μs (Figure 2C), leading to binding of the C2 domain to the membrane, with no subsequent dissociation over the course of the 2-μs simulation.

**Figure 3:**
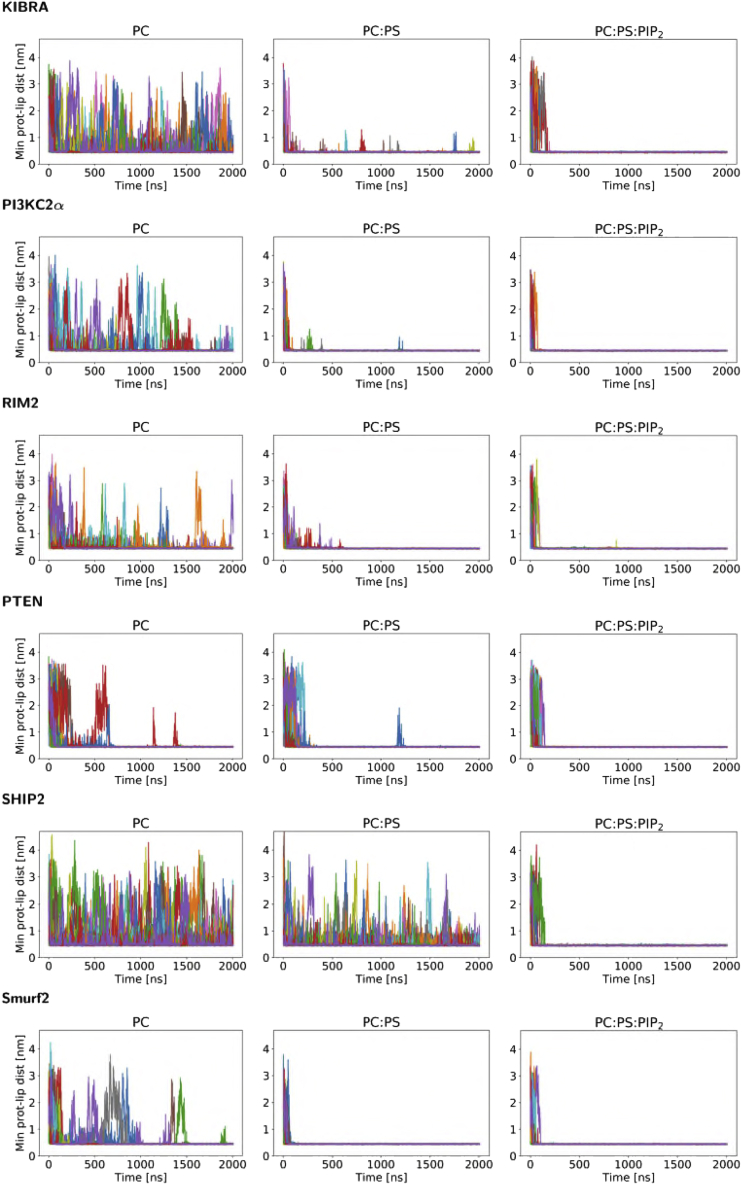
Minimum protein-lipid distances as a function of time for all simulations For each simulation ensemble the minimum protein-lipid distance is shown as a function of time, with the different colors corresponding to the 25 repeats within the ensemble. A distance of <0.5 nm corresponds to a contact between the protein and the lipid bilayer. For each C2 domain, simulations are shown for interactions with a PC, a PC:PS, and a PC:PS:PIP_2_ bilayer. See also Table S1; Figures S1 and S2.

We repeated this analysis for the 25 repeats for all six C2 domains and three lipid compositions (Figure 3). The variation among repeats is relatively large, especially for simulations with PC bilayer. Such stochastic behavior is, however, expected for single-molecule events, and the overall pattern seen for the Smurf2-C2 is conserved for all C2 domains: the simulations with a PC bilayer lead to multiple reversible contacts between the C2 and the membrane. For the anionic PC:PS bilayer, in most cases multiple encounters are seen, on average leading to a longer-lasting interaction of the protein and the membrane. In contrast, in the presence of a PC:PS:PIP_2_ bilayer, in all cases the initial encounter of C2 domain and bilayer leads to a protein/membrane interaction that persists for the remainder of the simulation. These simulations, especially those with PC:PS, enable us to come up with an initial, approximate ranking of the strength of interactions of C2 with a bilayer. Thus, comparing two C2 domains from PIP phosphatases (i.e., PTEN and SHIP2), we may contrast PTEN-C2, which on average forms long-lasting interactions with both PC and PC:PS bilayers, with SHIP2-C2, which forms multiple reversible interactions with both PC and PC:PS bilayers and only forms long-lasting interactions with a PC:PS:PIP_2_ bilayer. Fitting exponential decays to the averaged minimum protein-lipid distance versus time data for these simulations (Figure S1 and Table S1) supports this interpretation.

**Figure 4:**
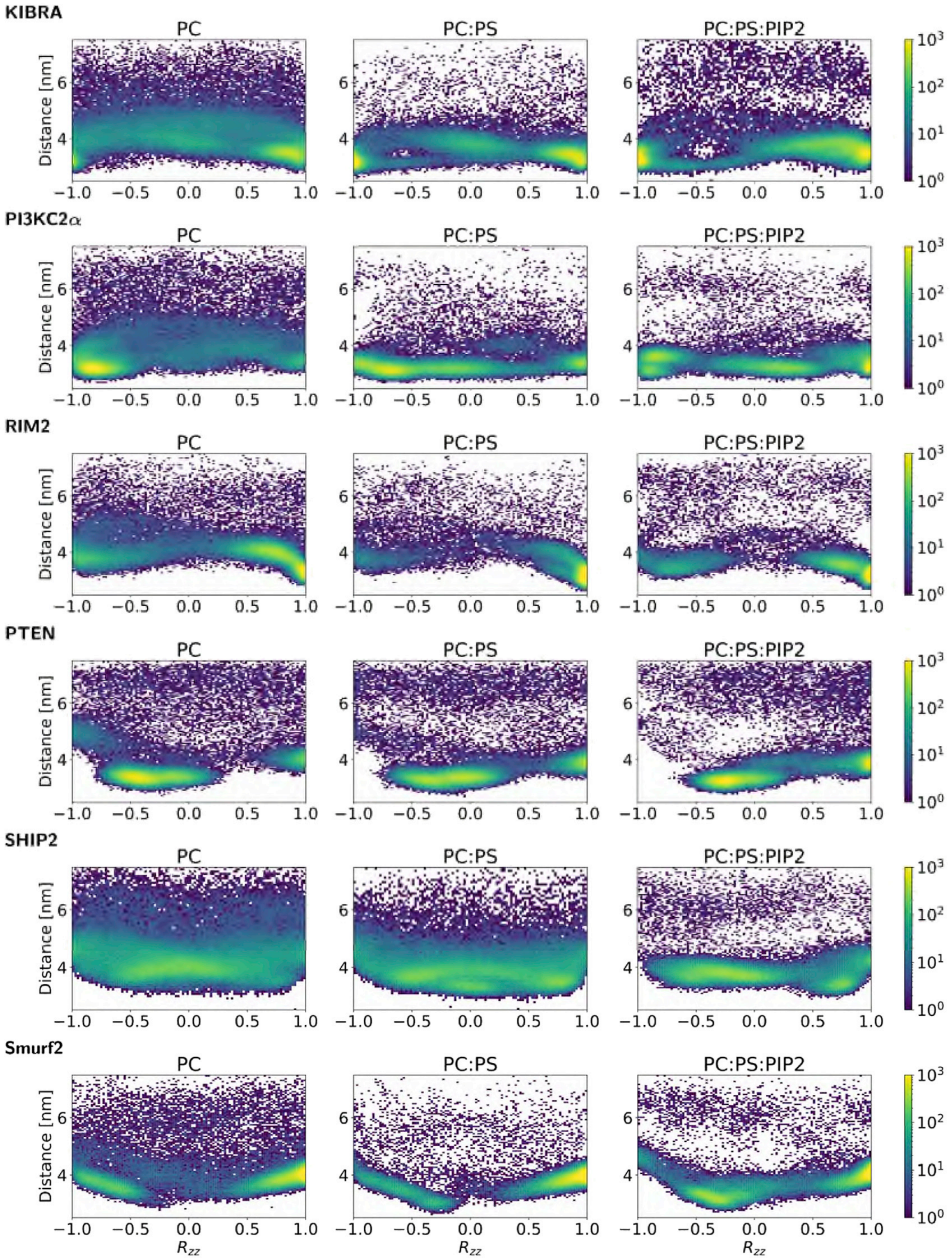
Density maps of the orientation and distance of the C2 domains relative to the lipid bilayer For each simulation ensemble, a density map of the orientation and distance of the C2 domain relative to a lipid bilayer is given. Each density map represents the relative frequency (on a logarithmic color scale from purple [low] to yellow [high]), averaged across time and all 25 simulations in an ensemble, of the orientation and distance of the C2 domain relative to the bilayer. The orientation is given by *R*_*zz*_, which is the *zz* component of the rotation matrix of the C2 domain with respect to a reference structure at *R*_*zz*_ = 1 (see text for details of the reference structure). Note that *R*_*zz*_ = −1 means that the protein is rotated 180° with respect to the reference structure. The distance shown is the *z* component of the vector between the centers of mass of the bilayer and the C2 domain. See also Figure S3.

The mode(s) of binding of the C2 domains were characterized by calculation of protein-lipid distance versus orientation density maps, which is an approach previously adopted for comparing PH domains (Yamamoto et al., 2016). The orientation of the C2 domain relative to the bilayer was quantified as the *zz* component of the rotation matrix, *R*_*zz*_, with respect to a reference frame (Figure 4). Note that *R*_*zz*_ = 1 means that the orientation along *z* is the same as the reference, whereas a value of −1 means that the protein is rotated 180°. (We only consider this one component of the orientation, as the lipid bilayer is isotropic in the *xy* plane.) The last frame of the first repeat of the ensemble was used as a reference frame in the initial calculation of *R*_*zz*_, and subsequently all *R*_*zz*_ values were recalculated with respect to a selected primary binding mode in the system with PC:PS:PIP2 bilayer once this mode was determined. The same reference was used for the systems with PC and PC:PS bilayers. We found multiple binding modes for the isolated C2 domains, showing up as dense areas in the distance/orientation maps (Figure 4). In most cases, the modes were the same for zwitterionic membrane (PC), anionic membrane (PC:PS), and anionic membrane with PI (PC:PS:PIP_2_), and the most visited mode was in many cases the same for all membrane compositions (Figure 4). However, the C2 domains spend more time unbound and away from the membrane for the PC membrane, while the presence of PS and PI enhanced membrane-protein contacts.

One primary binding mode was selected for each C2 domain (two for PI3KC2α). The selection criteria were: (1) the selected mode was probable, i.e., that the mode was frequently visited; (2) the mode was physically reasonable, i.e., that domains adjacent to the C2 domain in the intact protein would not overlap with the membrane; and (3) the mode was productive, i.e., adjacent catalytic domains were in contact with the bilayer. Interestingly, some modes are intermediates, as can be seen by monitoring each trajectory and following the path toward the final binding mode. Comparable dynamic behavior has previously been observed for other membrane binding proteins (e.g., K-Ras4A [Li and Buck, 2017]). In the current study we have, however, chosen to focus on the primary binding mode(s) rather than the details of the interaction pathway.

We evaluated the possible sensitivity of our results to changes in the lipid bilayer composition by systematic changes in the latter, using the PTEN C2 domain as a test case. We selected the PTEN domain because it had been seen to bind in two modes and had already been shown to exhibit some degree of sensitivity to changing the bilayer from zwitterionic (PC) to anionic (PC:PS) to anionic with PI (PC:PS:PIP_2_) (Figures 3 and 4). We therefore further explored the effects of: (1) increasing the fraction of PS from 20% to 40%; (2) replacing PIP_2_ by PIP or PIP_3_; and (3) inclusion of cholesterol alongside PC, PS, and PIP_2_. The results of these simulations are shown in Figures S2 and S3. These data do not suggest any major effects of varying the anionic lipids present, which is encouraging in that it shows our basic result of PIP_2_ promoting C2 binding is robust to changes. Notably, the simulations suggest that binding of the C2 domains is relatively insensitive to a change from, e.g., PIP_2_ to PIP_3_, at least in the CG simulations, despite PIP_3_ being the substrate for PTEN. This is broadly consistent with experimental studies, as reviewed by, e.g., Harishchandra et al. (2015), which indicate comparable binding affinities for PIP_2_ and PIP_3_. Perhaps surprisingly, given *in vitro* experimental data suggesting PTEN binding is not altered by the presence/absence of cholesterol (Das et al., 2003), the inclusion of 25% cholesterol speeds up binding of the C2 domain. This effect is partly due to a reduction of the area per lipid, resulting in increased surface charge density (Doktorova et al., 2017) (Figure S1). Distance/*R*_*zz*_ analysis (Figure S3) suggests that the modes of binding in the presence of cholesterol are very similar to those in the corresponding PC/PS/PIP_2_ simulations. The acceleration of C2 binding by cholesterol is intriguing and will form the subject of a subsequent more detailed study of lipid effects on C2 domain interactions.

### Two views of the energetics of C2/membrane-lipid interactions

To quantitatively compare the binding modes of the different C2 domains, we calculated binding free energies for each system. First, a potential of mean force (PMF) was calculated using umbrella sampling with the center-of-mass distance between protein and membrane as reaction coordinate (Figure 2D). This provides an estimate of a free energy curve for the interaction of the protein with the membrane. The C2 domains bound most strongly to PC:PS:PIP_2_ membrane, less tightly to anionic membrane without PIP_2_ (PC:PS), and even more loosely to PC membranes (Table 1 and Figure S4). The binding strengths had approximate ratios of 1:2:4 to 1:2:5 across the three membranes (PC versus PC:PS versus PC:PS:PIP_2_, Table 1).

Next, free energy perturbation (FEP) calculations provided additional information on the binding strength of specific PIP_2_ lipid head groups with the C2 domain (Figure 2E and Table 2). Four PIP_2_ molecules were bound to each C2 domain, with free energies ranging from −4 to −21 kJ/mol for each PIP_2_ molecule. Generally, the binding energies were evenly distributed among the four PIP_2_ head groups bound in each system (Table 2).

**Table 2:** Interaction free energies of PIP_2_ head groups

Domain	Binding mode	FEP for each of the four bound PIP_2_ molecules^a^ (kJ/mol)	Total FEP (kJ/mol)	ΔPMF^b^ (kJ/mol)
KIBRA-C2	side B	6, 7, 7, 6	26.0 ± 3.9	22.0 ± 2.1
PI3KC2α-C2b	front	9, 7, 10, 7	33.3 ± 4.0	35.5 ± 1.8
	back	10, 7, 4, 10	30.9 ± 4.1	72.4 ± 2.7
RIM2-C2a	side A	11, 21, 15, 12	58.0 ± 3.6	70.6 ± 2.6
PTEN-C2	top	10, 7, 9, 7	32.8 ± 4.0	50.7 ± 5.0
SHIP2-C2	top	4, 4, 4, 4	15.7 ± 2.7	12.7 ± 1.3
Smurf2-C2	top	6, 6, 9, 8	27.9 ± 4.4	43.3 ± 2.5

a Energy calculated by changing the head group of each PIP_2_ in the upper leaflet to PC while keeping the other PIP_2_ molecules unaltered. Mean of five repeats. Error on the total FEP is calculated by the standard deviation from the five repeats.

b PMF well depth difference between binding to PC:PS (80:20) and binding to PC:PS:PIP_2_ (80:15:5). See also Figures S5–S7.

As an internal check, we calculated binding energies via both the PMF and FEP (Figures 2D and 2E). Since all PIP_2_ in the upper leaflet were bound to C2 and these were converted to PC, the upper leaflet was effectively converted from a PC:PS:PIP_2_ (80:15:5) membrane to a PC:PS (85:15) membrane. Therefore, the FEP energies should be similar to the energy difference between the PMF for C2 bound to PC:PS:PIP_2_ (80:15:5) membranes and C2 bound to PC:PS (80:20) membranes (ΔPMF, Table 2). The agreement is not perfect, but the trends are the same and absolute values are close (Table 2). Binding energies were always smallest for binding to PC membranes, larger for PC:PS binding, and largest for PC:PS:PIP_2_ membranes. A notable exception is that ΔPMF is significantly larger than FEP for PI3KC2α. This is a general trend for C2 domains with large absolute binding PMF (compare Tables 1 and 2). For systems with small absolute binding energy, on the other hand, there is no such discrepancy.

### Binding modes, AT-MD, and comparison with experimental data

**Figure 5:**
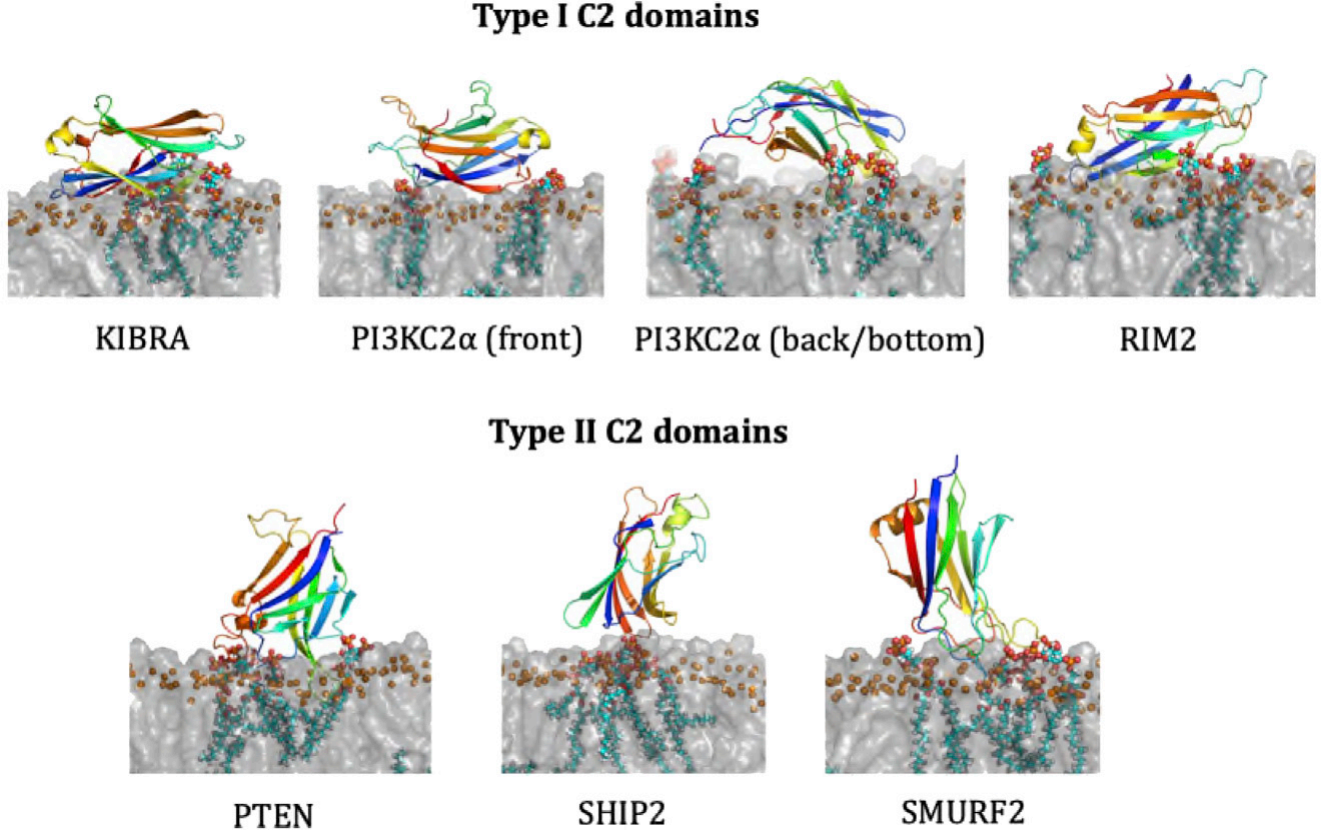
C2 membrane binding modes from atomistic simulations Snapshot of the C2 domains at the end of 200-ns atomistic simulations while bound to a PC:PS:PIP_2_ bilayer. POPC (1-palmitoyl-2-oleoyl-*sn*-glycero-3-phosphocholine) and POPS (1-palmitoyl-2-oleoyl-*sn*-glycero-3-phospho-L-serine) are depicted as a transparent gray surface with their P atoms highlighted as orange spheres, and PIP_2_ is shown as van der Waals spheres. The C2 domains are shown as cartoon representations on a rainbow color scheme from the N terminus (blue) to the C terminus (red). See also Figure S8.

In the last step of the protocol, each system was converted from coarse-grain resolution to atomistic resolution, and a 200-ns simulation was performed (Figure 2F) to refine the binding modes (Figure 5). All C2 domains with type II topology bound with the top facing the membrane (Figure 5 and Table 1). The C2 domains with a type I topology, on the other hand, bound with either the side, front or back, toward the membrane (Figure 1), i.e., with its longest axis parallel to the membrane. This is reflected in the PMF calculations through the distance with the minimum binding energy; the type I C2 domains have lowest energy at a center-of-mass distances between 3.1 and 3.5 nm, whereas the type II C2 domains have lowest energy at distances between 3.6 and 4.0 nm, for the productive mode (Table 1). In all simulations with PIP_2_ present, the PIP_2_ molecules clustered around the C2 domain (Figure 5) with all four PIP_2_s in the upper leaflet bound for most of the time.

The contacts between basic (Arg and Lys) side chains of C2 and the phosphates of PIP_2_ were analyzed for the binding modes observed in the atomistic simulations (Figure 5). Interestingly, there is a correlation between the number of contacts and the depth of the free energy well in the corresponding PMF (Figures S4 and S8; Table 1), indicating that electrostatic interactions between basic side chains of C2 and the PIP_2_ head groups are dominant but not the sole determinants of the interactions.

**Figure 6:**
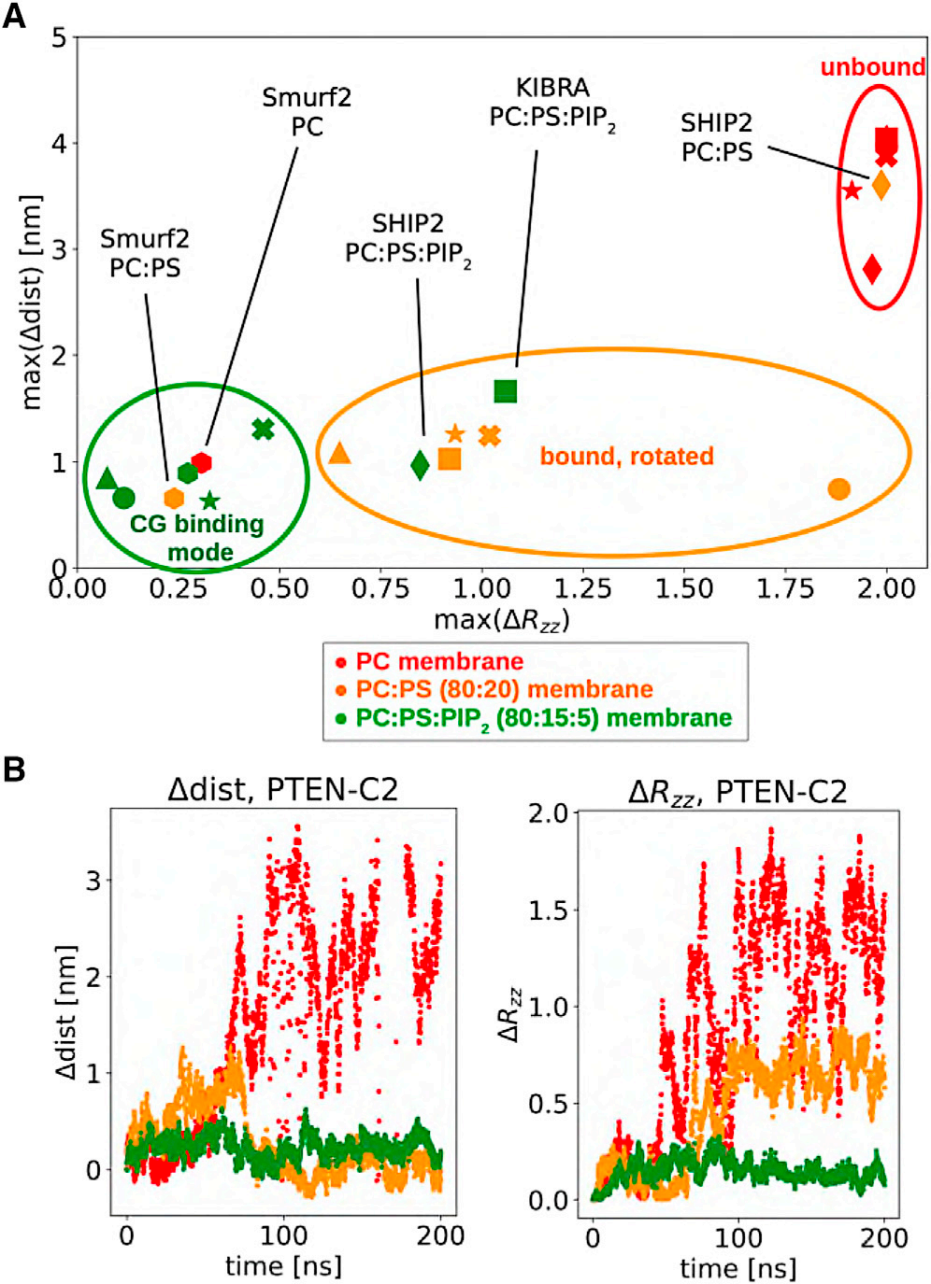
Changes in C2 orientation and distance relative the bilayer during atomistic simulations (A) The maximum changes in distance and orientation (see STAR methods) with respect to the initial frame (the CG binding mode) for the atomistic simulations. The simulations can be divided into three groups, shown by: a green ellipse, where the protein remains in the initial binding mode; an orange ellipse, where the protein remains bound but changes orientation (i.e., rotates relative to the bilayer); or a red ellipse, corresponding to those simulations where the C2 domain dissociates from the membrane. Different point styles correspond to different C2 domains (KIBRA [square], PI3K [front mode, circle], PI3K [back mode, cross], RIM2 [triangle], PTEN [star], SHIP2 [diamond], and Smurf2 [hexagon]), and point colors correspond to the lipids present in the bilayer (PC [red], PC:PS [orange], and PC:PS:PIP2 [green]). (B) An example of the change in distance and orientation during atomistic simulations of the PTEN-C2 domain initially bound to PC (red), PC:PS (orange), or PC:PS:PIP_2_ (green) bilayers.

The C2 domains generally stayed in their initial bound configuration with PIP_2_ throughout the atomistic simulation, whereas C2 domains bound to PC:PS rotated more and unbound in one case, and C2 domains bound to pure PC membranes unbound in all but one case (Figure 6). The conformation of the proteins did not change, with Cα-root-mean-square deviations not exceeding 0.3 nm as compared with the crystal structure for any of the C2 domains. This justifies the use of an elastic network applied to the proteins in the CG simulations, which preserves secondary structure and tertiary fold.

In the following, we compare the binding modes observed in the simulations with structural and other experimental data where available.

#### Smurf2

The N-terminal C2 domain localizes Smurf (SMAD-Specific E3 Ubiquitin Protein Ligase 2) to the membrane (Feng and Derynck, 2005; Kavsak et al., 2000; Wiesner et al., 2007). Smurf2-C2 showed one dominant binding mode in our CG simulations, which is the same for all three membranes (Figure 4). This is a canonical top binding mode with loops L12, L56, and L78 facilitating lipid binding (Figure 5). These loops were previously determined as phospholipid binding sites by nuclear magnetic resonance (NMR) (Wiesner et al., 2007). Weisner et al. further determined that Smurf-C2 binds PIP_1_, PIP_2_, and PIP_3_ by lipid overlay assays. The Smurf-C2 stays in the same binding mode during 200-ns atomistic simulations for all three membranes (Figure 6). Notably, Smurf-C2 is the only protein out of the six investigated that stays bound to the PC membrane throughout the atomistic simulations. Smurf2-C2 shares 87% sequence identity with the C2 domain of Smurf1 (Figure S5), and they have similar functions and structure (Koganti et al., 2018), which make it relevant to compare them. Scott et al. (2020) used MD simulations to explore interactions of InsPs with Smurf1-C2 and observed a canonical top binding mode, in line with our results. Moreover, Smurf1-C2 has been crystallized with negatively charged sulfates bound, also on the top of the C2 domain (PDB: 3PYC; Figure S5).

#### KIBRA-C2

Kidney and brain expressed protein (KIBRA) is a multi-functional protein with around 20 known binding partners and is central in many cellular processes, e.g., membrane trafficking (Milnik et al., 2012). KIBRA contains a C2 domain, which facilitates membrane binding in a Ca^2+^-independent manner (Posner et al., 2018). KIBRA-C2 displays two dominant binding modes in our simulations, but the most frequent binding mode is with β7 and β8 facing the bilayer (Figure 5), i.e., with side B (Figure 1) toward the lipid surface. This binding mode is consistent with previous NMR, X-ray crystallography, and MD data for the lipid-binding site of KIBRA-C2 (Posner et al., 2018), as it involves β1 and β8 and binds in a non-conventional way (i.e., not via the not loops). The second binding mode (Figure 4) is unphysical, as the N and C termini are buried in the bilayer and KIBRA-C2 is a central domain. During the 200-ns atomistic simulation, the C2 domain changed orientation slightly so the front faced the membrane (Figure 6) when bound to either PC:PS or PS:PS:PIP_2_ (the location of the front is shown in Figure 1). This mode is, however, still consistent with experimental data, as it involves β1, β2, and β8 in its lipid association. When initially bound to a PC membrane, the C2 domain diffused away from the bilayer during the AT simulation.

#### RIM2-C2a

RIM2 (Rab3 Interacting Molecule 2) is involved in synaptic vesicle priming (Dai et al., 2005; Südhof, 2004). It contains two C2 domains, one in the middle of the protein (C2a) and one in the N terminus (C2b). C2a was studied here. RIM2-C2a has a dominant binding mode in our simulations, with the front of the C2 domain binding the membrane (Figure 5) for all three membranes (Figure 4). A large binding energy to membranes for RIM2-C2 compared with the other C2 domains (Table 1) is likely due to a highly polar surface, with a positive patch on side A (β7 and β8) and a negative patch on side B (β4 and β5) (Dai et al., 2005), resulting in strong binding between side B and negatively charged membranes. RIM2 C2 has one particularly strongly bound PIP_2_ (with an FEP energy of 21 kJ/mol; Table 2). This lipid is bound to β4 of RIM2-C2, suggesting a specific PIP_2_-binding pocket at this location. In the atomistic simulations, the protein retained its orientation with a PIP_2_-containing membrane, underwent a rotation with PC:PS, and dissociated from the PC bilayer (Figure 6). RIM2-C2a has been crystallized with sulfates, which were suggested to bind at the same location as negatively charged PIP head groups (PDB: 2BWQ) (Dai et al., 2005). These sulfates are bound at a positively charged patch at the bottom of RIM2-C2a (Figure S6). In our simulations, a PIP_2_ head group was also bound at this site (Figure S6) (with binding energy of 11 kJ/mol; Table 2), suggesting that this is a specific PIP_2_-binding site.

#### PI3KC2α-C2b

PI3K (phosphoinositide 3-kinase) has a central C2 domain (C2a) and a C-terminal C2 domain (C2b). The structure of C2b has been solved by X-ray crystallography (PDB: 6BU0; note that C2b is referred to as PI3KC2α_C2C_ in Chen et al., 2018). C2b along with the adjacent C-terminal PX domain autoinhibit kinase activity in solution, but, interestingly, PX and C2b increase enzymatic effect at PI(4,5)P_2_-containing membrane (Wang et al., 2018). In our simulations, PI2KC2α-C2b revealed two dominant binding modes in our simulations with PC:PS:PIP_2_ membranes, with, respectively, the front and the back of PI3K-C2b facing the membrane (Figure 5). In the front binding mode, the lipids bind PI3K-C2 mainly via the C-terminal β strand, β8. In the back binding mode, the lipids mainly bind with β4 and the loops L34 and L67, but also via β7 and L23. Both binding modes are possible, as they do not block the neighboring PX domain in PI3KC2α, which is connected via a flexible loop at the N terminus of C2 (Chen et al., 2018). The crystallographic asymmetric unit of 6BU0 contains three copies of the C2b domain and four InsP_6_ molecules, which therefore define two distinct binding sites on the surface of the domain (Chen et al., 2018). The first binding site is at the front of the C2 domain and the second is at the back, with L34 and L67 contacting the bound InsP6 molecule (Figure S7). Thus, the simulations and the crystal structure both suggest these two binding modes, i.e., a front and a back binding mode. The front binding mode had four PIP_2_s bound in the simulations, but none of them coincided with the InsP_6_ from the crystal structure. Instead, the PIPs were bound close to the loops in the top and bottom of the C2 domain. The back binding mode likewise had all four PIP_2_s from the upper leaflet bound, one of which was bound in proximity to the binding pocket of the crystal, i.e., the PIP_2_ head group from the simulation was bound about 0.8 nm from the crystallographic InsP_6_ (Figure S7). This suggests that this may be a more specific binding pocket for PIP_2_, albeit having a relatively low binding affinity (4 kJ/mol; Table 2) compared with the other bound PIPs.

#### PTEN-C2 and SHIP2-C2

Both of these C2 domains form the C-terminal half of a core enzyme structure, made up of a catalytic phosphatase (Ptase) domain followed by a membrane-targeting C2 domain (Le Coq et al., 2017; Worby and Dixon, 2014). This enables us to identify which binding modes of the isolated C2 domains are “productive.” Also, the simulated binding interactions of the isolated C2 domains may be compared with those of the core Ptase-C2 structures.

PTEN is a tumor suppressor that dephosphorylates PIP_3_ to PI(4,5)P_2_ (Worby and Dixon, 2014). The C2 domain of PTEN acts by bringing the phosphatase domain of PTEN (Ptase) close to the membrane. The isolated C2 from PTEN exhibits two binding modes in our simulations (Figure 4). In the first mode, C2 binds with the loops in the top of the domain (Figure 5), whereas the second binding mode, which has the back of C2 interacting with the bilayer, is unphysical as the Ptase domain of PTEN would clash with the membrane, as seen by comparison with the X-ray crystal structure (Lee et al., 1999) (PDB: 1D5R). Therefore, the top binding mode is the only physical binding mode observed in our simulations, and this mode is also consistent with previous MD simulations of PTEN (Kalli et al., 2014). Electrostatics is the main driver component in the lipid binding, as has also been shown in binding studies of PTEN-C2 with different membranes (Das et al., 2003), but there is no simple linear correlation between bilayer surface charge density and membrane affinity (Figure S1), suggesting that electrostatics is not the sole component.

SHIP2 (SH2-containing-inositol-5-phosphatase 2) dephosphorylates PIP_3_ to PI(3,4)P_2_, and its affinity toward both PIP_3_ and PI(4,5)P_2_ has been demonstrated experimentally (Chi et al., 2004; Pesesse et al., 1998). SHIP2-C2 exhibits three distinct binding modes in simulations with a PC:PS:PIP_2_ membrane (Figure 4). The structure of SHIP-C2 has been determined with the adjacent Ptase domain (Le Coq et al., 2017) (PDB: 5OKM), and in two of the modes the Ptase domain is unproductively protruding out in the cytosol, with no contact with the lipids. In the productive mode Ptase is close to the membrane, facilitated by C2. This latter binding mode corresponds to the top loops binding to the membrane, in particular L12, L34, and L56 (Figure 5). Interestingly, the PMF for SHIP2-C2 reveals that it binds weakly (Figure S4) with a broad minimum encompassing all three binding modes. Furthermore, even when bound to the PIP_2_-containing membrane, SHIP2-C2 has some rotational freedom in the atomistic simulation (Figure 6).

### Simulations of larger fragments of PTEN and SHIP2

As noted above, for PTEN and SHIP2 the structures of the core proteins (Ptase-C2) are known, so it is possible to also simulate these and compare the modes of membrane interactions with those of the isolated C2 domains. There have been a number of previous simulation studies of the interaction of PTEN with membranes (Gericke et al., 2013; Kalli et al., 2014; Lumb and Sansom, 2013; Nanda et al., 2015; Shenoy et al., 2012a, 2012b; Treece et al., 2020), but we have simulated it here again to maintain the same simulation settings as for isolated C2, in particular the same lipid composition, namely PC:PS:PIP_2_ (80:15:5).

**Figure 7:**
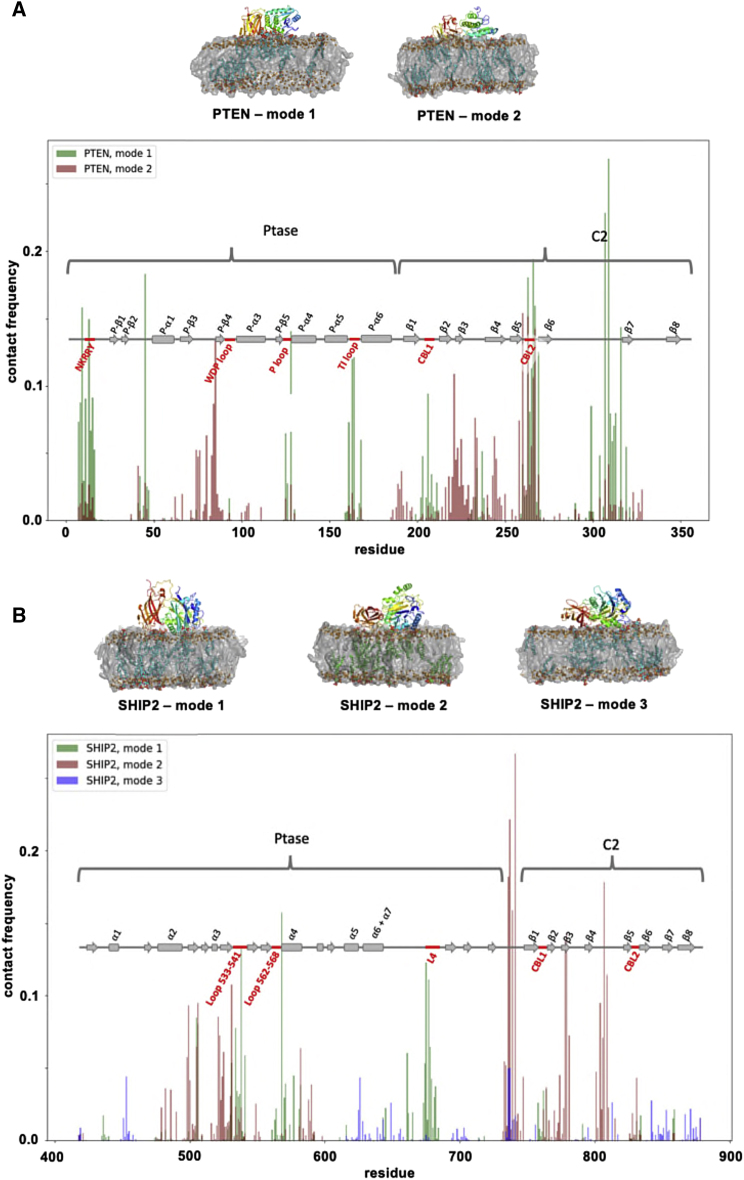
CG simulations of the interaction of intact PTEN and SHIP2 enzymes with PIP_2_-containing bilayers (A) Contact frequency to PIP_2_ for each residue of PTEN. The two modes of PTEN binding are shown in green (mode 1) and red (mode 2). The secondary structure of PTEN is shown as rectangles for α helices and arrows for β strands. The N-terminal domain, the WDP loop, the P loop, and the TI loop (red) are all important for phosphatase activity. (B) Contact frequency for each residue of SHIP2. Three modes of SHIP2 binding are shown in green (mode 1), red (mode 2), and blue (mode 3). The loop L4, the loop containing residues 533–541, and the loop containing residues 562–568 (red) are part of the phosphatase catalytic site.

As described above, for isolated PTEN-C2 two binding modes were observed, only one of which was “physical,” with the top loops pointing toward the bilayer. In the simulations of PTEN (Ptase + C2), we also obtained two binding modes (Figure 7A). One mode (∼50% of the population; mode 1 in Figure 7A) was comparable with the “physical” mode for the isolated C2 domain and allowed both the Ptase and C2 domains to contact the membrane (Figure 7A). An alternative mode 2 was also observed with side A of C2 pointing toward the lipids. This mode was not seen for the isolated C2. The catalytic site of Ptase of PTEN involves the P loop, the WDP loop, and the TI loop (Lee et al., 1999), as well as an N-terminal motif covering residues 12–16 (NKRRY) (Gericke et al., 2013). In our simulated mode 1, the PIP_2_ molecules are in frequent contact with the N-terminal motif, the P loop, and the TI loop, and the PIPs have some contact with the WDP loop (Figure 7A). In the simulated mode 2 there is also contact between the PIPs and the N-terminal motif and the three loops, but substantially less so (Figure 7A). Therefore mode 1, which was also found for the isolated C2 domain, is likely to correspond to the productive binding mode, enabling the association of the catalytic domain with the membrane.

For the isolated SHIP2-C2 domain, as discussed above, a rather weak multi-mode interaction was observed. For the larger fragment of SHIP2 (Ptase + C2) we observed three binding modes (Figure 7B). Of these, one mode (mode 1), similar to the productive mode for the isolated C2, accounted for 36% of the population. In SHIP2, three loops are part of the catalytic site: the loop containing residues 675–684 (denoted L4 by Le Coq et al., 2017), the loop consisting of residues 533–541, and the loop consisting of residues 562–568, as judged from available crystal structures (PDB: 5OKM and 4A9C) (Le Coq et al., 2017; Mills et al., 2012). Notably, in mode 1 of the simulations of SHIP2, the Ptase has frequent contacts between the bound PIP_2_s and these three loops of the active site (Figure 7B). In the two other modes, on the other hand, there is little or no contact between PIP_2_s and the active-site loops. Interestingly, in mode 1 the C2 seems to interact relatively weakly with the membrane, whereas the Ptase domain is more tightly bound and PIP_2_ molecules cluster more around Ptase than around C2 (Figure 7B). This suggests that while the C2 domain may facilitate SHIP2-membrane interactions, in this case the Ptase domain is perhaps more important for the membrane interaction of SHIP2.

Comparing PTEN and SHIP2, these observations would suggest that whereas C2 from PTEN does, to some extent, determine the binding mode of PTEN, C2 from SHIP2 is less important in determining the binding orientation of SHIP2 and only makes a weak contribution to the avidity of binding. This is consistent with C2 from SHIP2 being the weakest binding C2 domain in the study (PMF = 23 kJ/mol), whereas C2 from PTEN has a higher membrane affinity (PMF = 78 kJ/mol).

## Discussion

We have demonstrated the utility of a simulation pipeline for exploring the interactions of Ca^2+^-independent C2 domains with anionic lipid bilayer models of cell membranes. This has allowed us to address a number of key questions by comparing the interactions of six different species of C2 domain, namely whether there is a systematic correlation between lipid binding and topology, how C2 domains bind to PIP molecules in a membrane, and whether C2 domains bind multiple PIP molecules, thus contributing to the formation of anionic lipid clusters. Here, we review the implications of these findings in more detail and discuss limitations of the method.

### Possible limitations of the Martini force field

Using a CG force field allowed us to fully sample the encounter and binding to the lipid bilayer of the C2 domains in an unbiased manner. In these simulations, the size of the box does limit the membrane-bending modulus, i.e., we effectively assume a flat bilayer in our simulations. Bilayer curvature effects might also be worth investigating systematically, but we consider it beyond the scope of the current study. Using a CG force field does have some known limitations. Electrostatic interactions are only approximately described in the Martini force field, so we did not investigate, e.g., the effect of salt concentration and only added sufficient Na^+^/Cl^−^ ions to neutralize the system. Therefore, some electrostatic interactions may be overemphasized due to the absence of buffer-like charge screening. Some of the C2 domain/membrane binding energies are large (above 100 kJ/mol, Table 2), which has led to suggestions that the Martini force field may be too “sticky” (see, e.g., the discussions in Herzog et al., 2016; Javanainen et al., 2017). However, we have previously used the Martini force field to investigate PH domain binding to PIP-containing membranes, a system for which there is a reasonable body of experimental data (Naughton et al., 2016, 2018; Yamamoto et al., 2016). For that system, the energies were not overestimated compared with experimental values (but rather tended to be underestimated unless one allowed for multiple PIP molecules binding to each PH domain), which strengthens our justification for applying a comparable approach to the C2 system. We therefore suggest that the substantial binding energies observed are not an artifact from the force field. Moreover the C2 domains, once bound to PIP_2_-containing membrane, generally stay in their binding pose in the atomistic simulations (Figure 6). In the control simulations in which the protein was bound to a PC-only bilayer, the C2 domains in most cases dissociated from the bilayer during the atomistic simulations (Figure 6). Experimental data measuring membrane binding energies of the investigated C2 domains remains limited, and we hope that this study will stimulate future experimental binding studies.

### Non-canonical binding of C2 domains

C2 domains were first recognized as a Ca^2+^-dependent protein, with a canonical binding orientation involving the calcium binding loops at the “top” of the domain (Figure 1) (Corbalan-Garcia and Gómez-Fernández, 2014; Nalefski and Falke, 1996). Several studies have, however, expanded the possible modes to include binding without calcium, and other studies have suggested that C2 domains can bind in a non-canonical orientation (Posner et al., 2018). We have systematically categorized and analyzed some of these non-canonical binding modes. We found that all of the six C2. domains bound to anionic membranes in the absence of Ca^2+^ ions and that three of these proteins bound in non-canonical orientations via the front, back, or side of the β sandwich (Figure 5; consult Figure 1 for definitions of front, back, etc.). Notably, our results suggest a connection between topology and binding orientation, with type I C2 domains binding non-canonically and type II C2 domains binding canonically, i.e., via the top loops.

### PIP_2_ binding and clustering

Clustering of PIP_2_ was observed on inspection of the final binding modes of the C2 domain (Figure 5). Binding of multiple PIPs to a single domain has also been reported for PH domains (Yamamoto et al., 2016), so this may be a general phenomenon for lipid-associated protein domains. Most of the binding energies for single PIP_2_ molecules are relatively small (<10 kJ/mol; Table 2), which may explain the lack of co-crystallized structures with, e.g., inositol phosphates in the absence of the possibility of binding to multiple PIP_2_s in a membrane when in a crystal.

### Binding strength

We compared the binding strength of the C2 domains by different means. From the minimum distance versus time data, we obtain the ranking (strongest binding first):

(PTEN, PI3KC2α, and Smurf2) > (RIM2, KIBRA) > SHIP2.

From the PMF calculations:

(PI3KC2α, RIM2) > (Smurf2, PTEN) > (KIBRA, SHIP2).

Finally, from the FEP calculations:

RIM2 > (PI3KC2α, PTEN) > (Smurf2, KIBRA, SHIP2).

So the consensus is that PI3KC2α, PTEN, RIM2, and Smurf2 form strong interactions whereas KIBRA and SHIP2 form weaker interactions. However, we note that (in part due to the limitations of the CG approach) we have not explored either PIP_2_ versus PIP_3_ or PI(4,5)P_2_ versus PI(3,4)P_2_ in terms of the strength of binding interactions. A more detailed examination of the strength and specificity of binding of PIP species would require development of robust atomistic simulation estimates of the energetics of protein binding to lipids (see Pant and Tajkhorshid, 2020 for an example of this) and also a consideration of possible effects of ionization states of PIP head groups and the influence of bound cations (see, e.g., Bilkova et al., 2017; Slochower et al., 2013).

### Driving force for lipid binding

The main driving force for the binding of C2 to the membranes appears to be electrostatics (see, e.g., Figure S8). However, protein-lipid interactions can also reflect the geometry around the binding sites as seen, e.g., for K-Ras4A (Li and Buck, 2017). We have not made a systematic investigation of this but do not observe a simple correlation between surface charge density and binding of the PTEN C2 domain (Figure S2), which suggests that electrostatics is not the sole factor controlling C2 binding. The impact of cholesterol suggests that lipid ordering may also be of importance.

### Methodology

MD simulations allow for a systematic investigation of several related membrane-recognition proteins, providing direct comparison between them. This would in most cases be challenging to do experimentally for six or more proteins. CG-MD allows us to both monitor binding of domains to bilayers of differing lipid composition and undertake free energy calculations. The consistency observed for PMF and FEP calculations (Table 2) suggests that the energy calculations are converged. Convergence is an issue for energy calculation of protein-membrane binding, as shown for PMF calculations of PH domains binding to a PIP-containing membrane (Naughton et al., 2016, 2018) and PIP_2_ bound to Kir2.2 integral membrane protein (Corey et al., 2019). In both cases, 500- to 1,000-ns sampling was needed per umbrella window to obtain convergence (we used about 80 windows per protein/membrane combination in the present study). FEP needed 200–300 ns per window to obtain convergence for PIP_2_ bound to Kir2.2 (Corey et al., 2019) (we used 20 windows). These accumulated simulation times are not feasible with AT-MD, so we did the energy calculations before converting to atomistic resolution. The CG force field may not, on the other hand, describe the energies accurately, so experimental benchmarking will be needed in the future to verify the absolute values of the energetics calculations.

We believe that the established method is applicable to other membrane-recognition domains. Similar computational approaches have already been exploited for comparative studies of PH domains (Naughton et al., 2016; Yamamoto et al., 2016), and the method can be applied to other domains as long as a sufficient number of solved structures are available.

There are hundreds of C2 domains (Nalefski and Falke, 1996), and the six included here are therefore not representative for all. We decided to limit the scope to include only C2 domains that (1) are Ca^2+^-independent, (2) are interacting with the inner membrane leaflet, and (3) are solved structurally. This limits the number of possible C2 domains to include. However, as new structures are solved the methods can be applied to these and results compared with the present results, to obtain more certain knowledge about the binding orientation, affinity, and specificity of Ca^2+^-independent C2 domains.

There are only relatively limited experimental data on lipid-protein interactions of the Ca^2+^-independent C2 domains investigated. We hope that this study will stimulate further biophysical experiments. Of particular interest would be a systematic examination of C2 domains from different proteins under the same protocol, to provide a benchmark dataset for comparison with computational analysis.

### Conclusions

We have investigated six different Ca^2+^-independent C2 domains. These are all constituents of multi-domain proteins that interact with the cell membrane and play a key role in signaling. Using multi-scale MD, we investigated their binding modes, including binding orientation, binding affinity, and lipid specificity. We found that binding orientation and structural topology were related: type I C2 domains bound to the anionic membranes via the sides, front, or back of the β sandwich, whereas type II C2 domains bound via the loops at the top of the structure. Calculated binding free energies revealed significant binding differences between the six C2 domains, but these binding energies were not systematically correlated with the domain topology. Moreover, the domains, in general, only bound and stayed bound to negatively charged membranes, i.e., those containing PS and/or PIP_2_, and bound significantly more strongly to PIP_2_-containing membranes. PIP_2_ clustered around the C2 domains upon binding, which suggests that C2 lipid interaction generally involves binding of several clustered PIPs, as also observed for the related PH domain (Yamamoto et al., 2016). PS, on the other hand, did not cluster around the C2 domains, nor did PC. We have thus provided an overview of expected binding modes, binding affinity, and binding specificity for non-canonical C2 domains, which improves our overall understanding of the cellular roles of C2 domains.

Equally important, we have presented a transferable methodology that can be utilized for novel structures of C2 domains and can readily be expanded for investigations of other protein domains interacting with lipid bilayer models of cell membranes. Advances in both spatially resolved lipidomics (Tsuji et al., 2019) and simulations of complex membrane models (Marrink et al., 2019) combined with systematic simulation methodologies will in the future allow us to reliably predict patterns of protein/membrane recognition (Irvine et al., 2019).

## STAR★methods

### Key resources table

**Table:** 

REAGENT or RESOURCE	SOURCE	IDENTIFIER
**Deposited data**		

KIBRA C2 structure	Posner et al., 2018	PDB: 6FJD
PI3KC2α C2 structure	Chen et al., 2018	PDB: 6BU0
RIM2	Dai et al., 2005	PDB: 2BWQ
PTEN	Lee et al., 1999	PDB: 1D5R
Smurf2	Wiesner et al., 2007	PDB: 2JQZ
SHIP2 C2 structure	Le Coq et al., 2017	PDB: 5OKM

**Software and algorithms**		

GROMACS	Abraham et al., 2015	www.gromacs.org
PyMOL	maintained and distributed by www.schrodinger.com	https://pymol.org/
Modeller	Fiser et al., 2000	https://salilab.org/modeller/
Martini 2.2	de Jong et al., 2013	http://cgmartini.nl/
alchemical-analysis script	Klimovich et al., 2015	https://github.com/MobleyLab/alchemical-analysis
scripts for generating simulations	this study	https://github.com/andreashlarsen/Larsen2021-C2

### Resource availability

#### Lead contact

Further information and requests for resources should be directed to and will be fulfilled by the Lead Contact, Mark Sansom (mark.sansom@bioch.ox.ac.uk).

#### Materials availability

No unique reagents or materials were generated in this study.

#### Data and code availability

This study did not generate new software. The simulation trajectory datasets supporting the current study have not been deposited as a public repository for MDsimulation data does not yet exist. Coordinates of the models generated by this study (as representative frames from simulations revealing the interactions of C2 with PIP_2_ and with cholesterol) are available from the corresponding author on request. Details of deposited coordinates employed are provided in the key resources table. Scripts for running the simulations are available at github.com/andreashlarsen/Larsen2021-C2.

### Experimental model and subject details

No experimental models were used. The experimental data for the MD simulations consisted of the protein coordinate set (i.e. PDB files) as detailed in the key resources table.

### Method details

#### Coarse-grained molecular dynamics (CG-MD) simulations

CG-MD simulations were done using the Martini 2.2 force field (de Jong et al., 2013) and performed in GROMACS 2018.6 (Abraham et al., 2015). The input structures were truncated to only contain the C2 domain, using PyMOL (The PyMOL Molecular Graphics System, version 2.0 Schrödinger, LLC) and missing residues were added using Modeller (Fiser et al., 2000). The protein was coarse-grained using the Martinize script (de Jong et al., 2013) with the default elastic network to maintain the tertiary structure. The protein was positioned above a lipid membrane using the Insane script (Wassenaar et al., 2015) with box size 7x7x18 nm^3^. For the simulations with the larger fragments of PTEN or SHIP2, the box size was 12x12x24 nm^3^. The protein placed at a minimum distance of 4.4 nm from the membrane, which is four times the VDW and electrostatic cutoff distances of 1.1 Å. 10% antifreeze water was added (Marrink et al., 2007) and the system was neutralised with Na^+^ or Cl^-^ ions. The protein was rotated through a randomly selected angle relative to the bilayer to avoid any bias in binding orientation. Three different membranes were generated for each protein: PC, PC:PS (molar ratio 80:20) and PC:PS:PIP_2_ (80:15:5). For the small box size (simulations with isolated C2), there were 160-162 lipids in total, and 8 PIP_2_ in the PC:PS:PIP_2_ bilayers (four in each leaflet). For the large box size (simulations with larger fragments of PTEN or SHIP2), each bilayer contained 360 PC, 66 PS and 22 PIP_2_ molecules. We used the Martini lipid POP2 for PIP_2_ (López et al., 2013). The system was first minimised, then equilibrated using a restraint to ensure that the protein could only encounter the upper leaflet of the membrane. The restraint was set up using PLUMED UPPER_WALLS (Tribello et al., 2014) at a protein-lipid centre-of-mass distance of 7 nm and an energy constant of κ = 50 (internal units of code). Equilibration was run in the NPT ensemble with a semi-isotropic Berendsen barostat (Berendsen et al., 1984) and time constant of 14 ps to keep pressure at 1 bar, and at v-rescale temperature coupling applied separately to lipids, protein and solvent, with a time constant of 1 ps to keep the temperature at 323 K. Equilibration was run with 20 fs time steps for 10 ns, and production runs were run with the same settings, but with 35 fs time steps and for 2 μs. 25 repeats were made for each protein/membrane combination, with a new rotation, and setup of the system for each repeat.

#### Generating Distance vs. R_zz_ Plots

Distances between the centre of mass of the protein and the centre of mass of the lipids were calculated for each frame using *gmx distance* (after centering the protein in the box). A reference frame was needed for calculating the rotation matrix. One frame for each protein was selected from one of the 25 simulations with membrane PC:PS:PIP_2_. The protein from this frame was extracted, and used as reference protein, also for simulations with PC and PC:PS membranes. The rotation matrix was then calculated for each frame by comparing the protein in each frame with the reference protein using *gmx rotmat*, after fitting in the *xy* plane. R_zz_ is the zz component of the rotation matrix.

#### Potential of mean force (PMF) calculations

PMFs were calculated using umbrella sampling with the centre-of-mass distance between protein and membrane as reaction coordinate (Figure 2E). As preparation for the PMF calculations, two steered MD simulation was performed to generate starting frames for the umbrella sampling along the reaction coordinate, using the GROMACS pull code. In the first, the protein was pushed into the bilayer, and in the second, the protein was pulled away from the bilayer. The distance between the centre of mass of the protein and centre of mass of the lipids was restrained with a harmonic potential with force constant of 1000 kJ/mol/nm^2^ and a rate of 0.2 nm/ns in the *z*-direction (positive and negative direction for push and pull simulation, respectively). A position restraint with a force constant of 15,000 kJ/mol/nm^2^ was applied on the strongly bound PIP_2_ lipids to prevent them from being pulled out of the membrane with the protein. Frames were retrieved from the steered MD run every 0.05 nm until a centre-of-mass distance of 7.0 nm was obtained for the last frame (about 80 windows per system). Umbrella sampling was then performed by sampling each retrieved frames for 1 μs with a 2000 kJ/mol/nm^2^ position restraint on the protein, but with the restraint on PIP_2_ removed. The PMF was calculated using *gmx wham* (Hub et al., 2010), skipping the first 200 ns as equilibration, and using the bootstrap method (option *nBootstrap*) to get uncertainties on the PMF values.

#### Free energy perturbations (FEP)

Free energy perturbations were done as previously described (Corey et al., 2019, 2020), converting the Martini lipid POP2 (i.e. PIP_2_) to Martini lipid POPC (Figure 2F). The head group beads were gradually changed, in 20 steps, as controlled by a parameter λ. Each of the four POP2 Martini lipids in the upper leaflet was converted individually to POPC, with the others kept as POP2. To keep the system neutral at all times, we gradually converted five sodium ions to neutral beads. Each frame was sampled for 1 μs, and the free energy was calculated using the *alchemical-analysis* script (https://github.com/MobleyLab/alchemical-analysis) and the MBAR method (Klimovich et al., 2015). The perturbation energies were also calculated for conversion of POP2 in a bilayer without bound protein. The reported energies are the difference between FEP energies for free and bound POP2 (Corey et al., 2019). Five repeats were made for calculation of uncertainties via the standard deviation.

#### Atomistic molecular dynamics (AT-MD) simulations

Selected frames with a membrane-bound C2 molecule were converted from CG (force field Martini 2.2) to AT (force field CHARMM36m (Best et al., 2012; Huang et al., 2017) and TIP3P water) using the CG2AT script (Vickery and Stansfeld, 2021). Atomistic simulations were run in GROMACS 2018.6 (Abraham et al., 2015). The system was first minimised, then equilibrated for 100 ps in the NVT ensemble and 100 ps in the NPT ensemble. The equilibrated system was run for 200 ns. Both equilibration steps and production run had 2 fs timesteps with 1.2 nm VDW and electrostatic cutoff distance, v-rescale temperature coupling keeping the temperature at 300 K with timeconstant of 0.1 ps, separately for protein, lipids and solvent. A Parrinello-Rahman barostat (Parrinello and Rahman, 1981) was applied to the NPT equilibration and production run with a semi-isotropic pressure coupling, keeping pressure at 1 bar with time constant of 5 ps and water compressibility of 4.5⋅10^-5^ bar^-1^. Long-range electrostatics were handled using the particle mesh Ewald method (Darden et al., 1993).

### Quantification and statistical analysis

Statistical analysis details can be found in the relevant sections of the STAR methods and the table legends. In particular, PMF well depths (Table 1) and FEP errors were calculated as the mean plus/minus the standard deviation as detailed in the STAR methods above.

### Additional resources

No additional resources were generated by this study.

## Supplemental information

Document S1. Figures S1–S8 and Table S1

Document S2. Article plus supplemental information
