# Supplementary material for: Binding of Ca2+-independent C2 domains to lipid membranes: A multi-scale molecular dynamics study
Source: Structure. 2021 Oct 7;29(10):1200–1213.e2. doi: 10.1016/j.str.2021.05.011 (PMC8507603; doi:10.1016/j.str.2021.05.011)
Supplement: Document S1. Figures S1–S8 and Table S1 [file mmc1.pdf]

Structure, Volume *29*

Supplemental Information

Binding of Ca^{2+}-independent C2 domains to lipid membranes: A multi-scale molecular dynamics study

Andreas Haahr Larsen and Mark S.P. Sansom

Supplemental Information for

Binding of Ca^{2+}-Independent C2 Domains to Lipid Membranes: a Multi-Scale Molecular Dynamics Study

*Andreas Haahr Larsen & Mark S.P. Sansom**

Department of Biochemistry, University of Oxford,

South Parks Road, OX1 3QU, Oxford, UK.

*Correspondence to *mark.sansom@bioch.ox.ac.uk*

Table S1. C2 domain distance vs. time exponential decays. The average decay curves derived from the protein-lipid minimum distance data in Fig. 3 were fitted with $D(t) = D_0 \exp(-\lambda t) + B$, where D is the average distance over the repeats, D_0 is the initial distance, λ is the decay rate, t is the simulation time, and B is the average of the final minimum distance for all repeats. Mean and SD are mean and standard deviations for a specific lipid over all the different C2 domains. See Fig. S1 for an example. Related to Figure 3.

Protein C2	PC		PC:PS		$PC:PS:PIP_2$	
	λ (ps^{-1})	B (nm)	λ (ps^{-1})	B (nm)	λ (ps^{-1})	B (nm)
Smurf2	22	0.47	53	0.45	32	0.45
RIM2	29	0.48	43	0.45	33	0.45
KIBRA	34	0.53	70	0.46	19	0.46
PTEN	15	0.46	13	0.56	19	0.45
SHIP2	38	0.59	44	0.51	30	0.46
PI3KCα	23	0.48	68	0.45	44	0.45
Mean ± SD	27 ± 8	0.50 ± 0.05	48 ± 19	0.48 ± 0.04	30 ± 9	0.45 ± 0.01

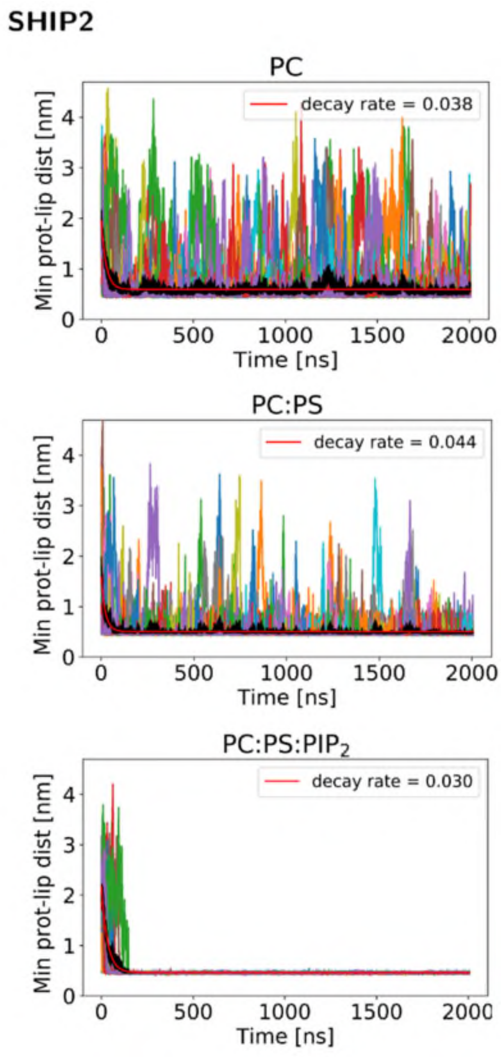

Fig. S1. Minimum protein-lipid distances as a function of time for the SHIP2 C2 domain, fitted with exponential decay curves. The data are as shown in Fig. 3. See Table S1 for details of the decay curves (red) fitted to the averaged data (black line). Related to Figure 3.

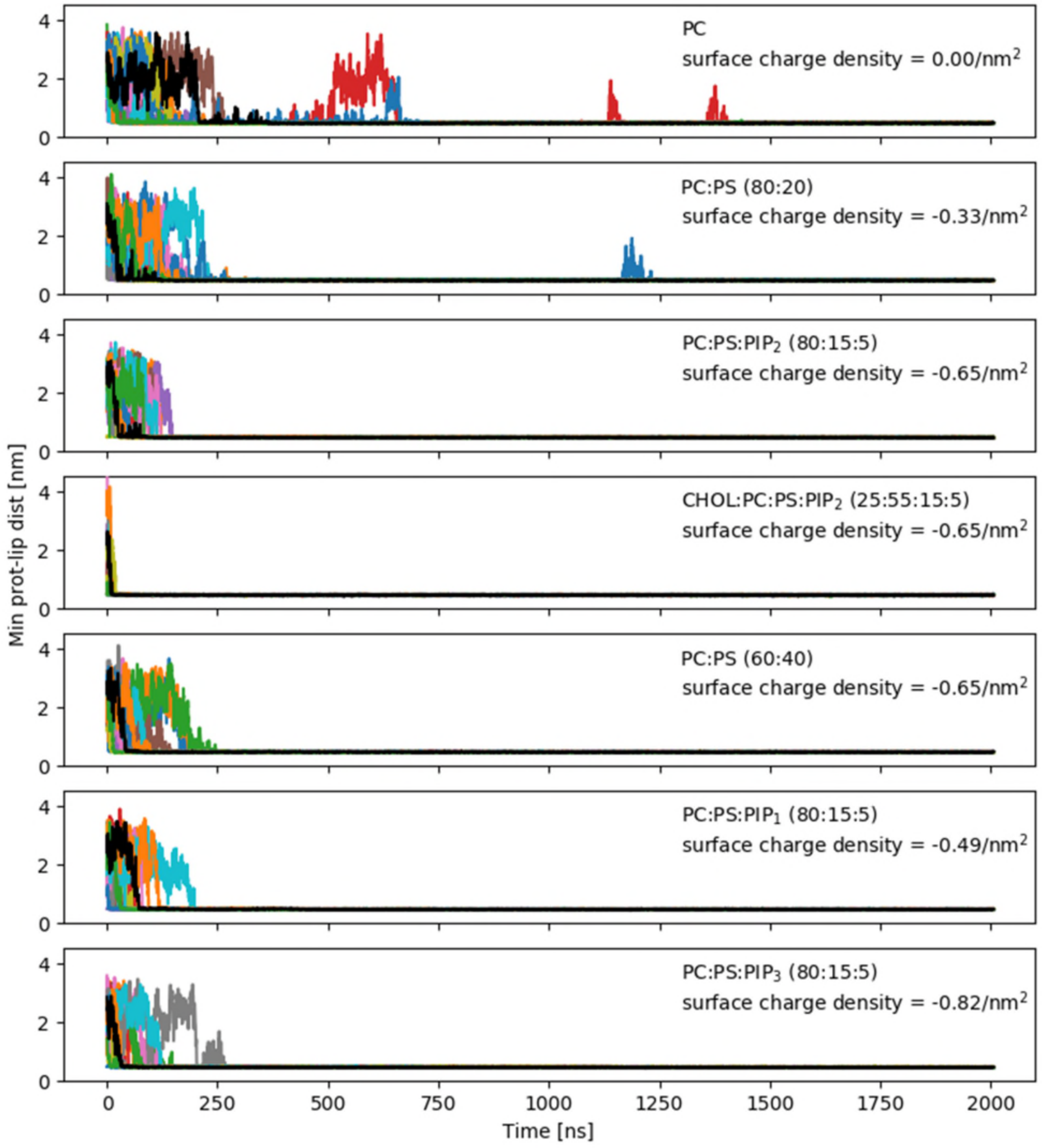

Fig. S2. Minimum protein-lipid distances as a function of time for PTEN C2 simulations with different bilayer lipid compositions. For each simulation ensemble the minimum protein-lipid distance is shown as a function of time, with the different colours corresponding to the 25 repeats within the ensemble. A distance of < 0.5 nm corresponds to a contact between the protein and the lipid bilayer. Related to Figure 3.

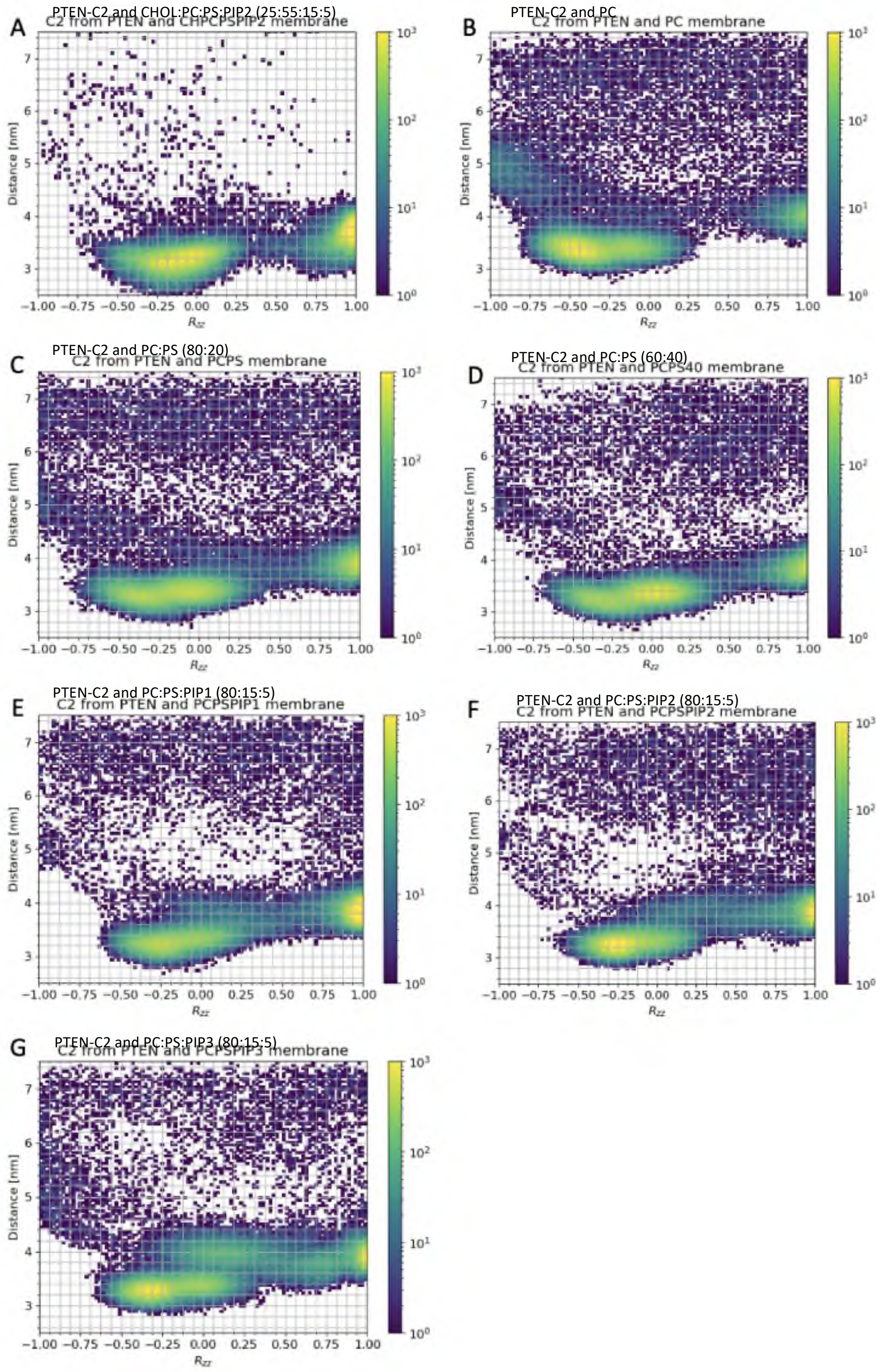

Fig. S3. Density maps of the orientation and distance of the PTEN C2 domains relative to bilayers with different lipid compositions. For each simulation ensemble a density maps of the orientation and distance of the C2 domain relative to a lipid bilayer is given. Each density map represents the relative frequency (on a logarithmic colour scale from purple low to yellow high), averaged across time and all 25 simulations in an ensemble, of the orientation and distance of the C2 domain relative to the bilayer. The orientation is given by R_{zz} which is the *zz* component of the rotation matrix of the PTEN C2 domain with respect to a reference structure at $R_{zz} = 1$ (see the main text for details of the reference structure). Note that $R_{zz} = -1$ means that the protein is rotated 180° with respect to the reference structure. The distance shown is the *z*-component of the vector between the centres of mass of the bilayer and the C2 domain. Related to Figure 4.

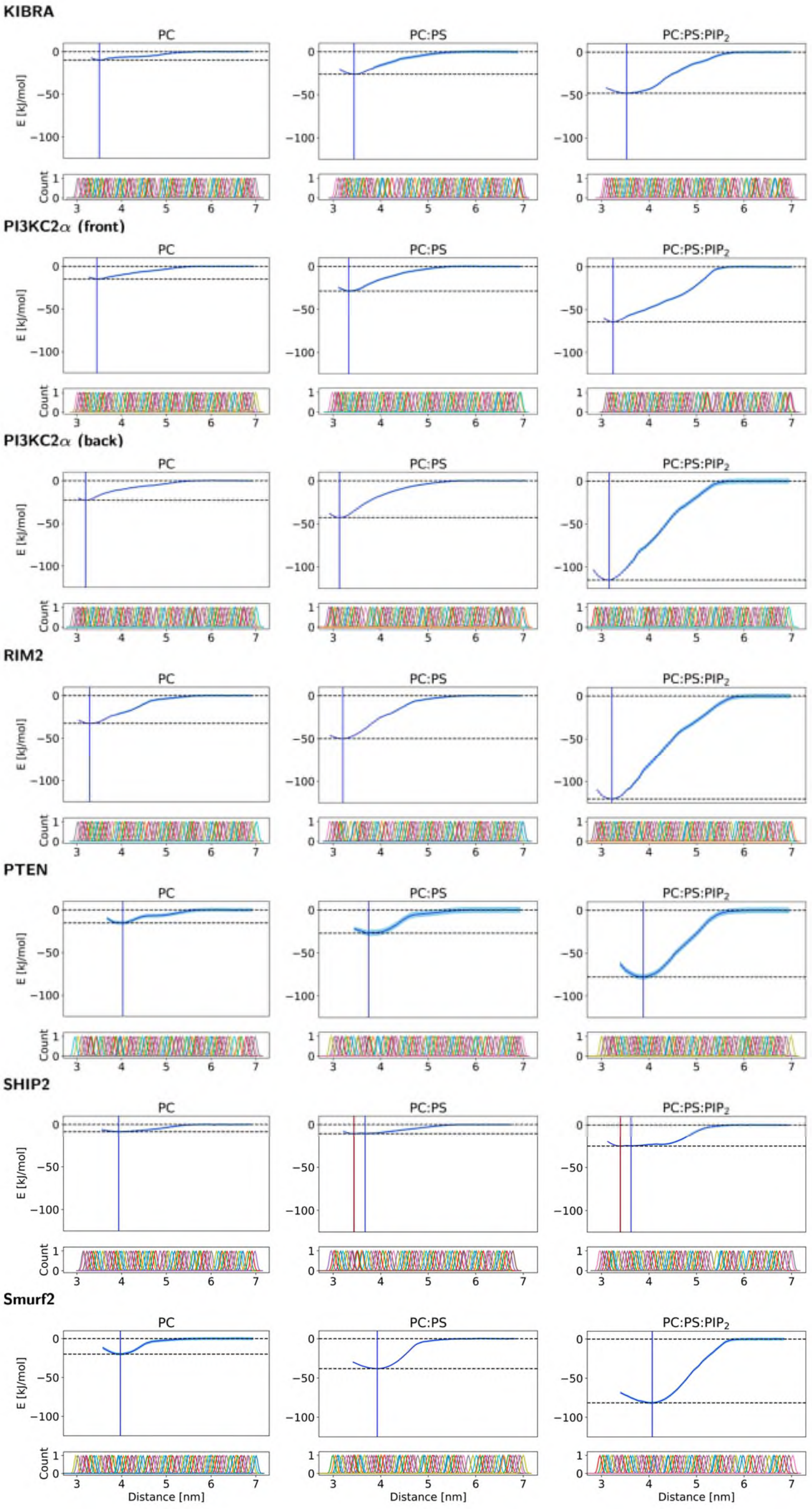

Fig. S4. Potentials of mean force for C2 domain/membrane interactions. For each C2 domain and bilayer, the potential of mean force (PMF) as calculated via umbrella sampling is shown, with the reaction coordinate corresponding to the distance between the centre of mass of the protein and centre of mass of the lipids. The dark blue curve shows the PMF and the pale blue area corresponds to ± one standard deviation. The two broken horizontal lines define the depth of the energy well corresponding to the minimum of the PMF. The corresponding histograms from the individual umbrella sampling window are shown beneath the PMF in order to demonstrate overlap. Related to Table 1.

A

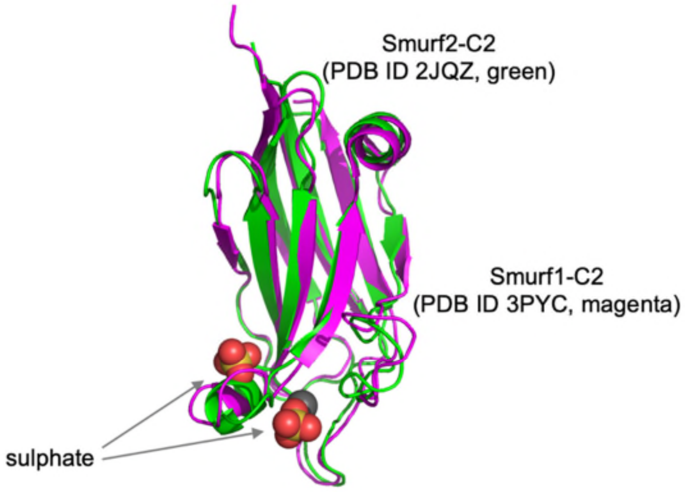

B

```
Alignment of 2JQZ (Smurf2-C2) and 3PYC (Smurf1-C2)
NW score    Identities          Positives    Gaps
615    116/133 (87%)      125/133      3/133 /2%)


2JQZ  1    GP--VKLRLTVLCAKNLVKKDFFRLPDPFAKVVVDGSGQCHSTDTVKNTLDPKWNQHYDL  58
           G   +K+RLTVLCAKNL KKDFFRLPDPFAK+VVDGSGQCHSTDTVKNTLDPKWNQHYDL
3PYC  1    GSEFIKIRLTVLCAKNLAKKDFFRLPDPFAKIVVDGSGQCHSTDTVKNTLDPKWNQHYDL  60

2JQZ  59   YIGKSDSVTISVWNHKKIHKKQGAGFLGCVRLLSNAINRLKDTGYQRLDLCKLGPNDNDT  118
           Y+GK+DS+TISVWNHKKIHKKQGAGFLGCVRLLSNAI+RLKDTGYQRLDLCKL P+D D
3PYC  61   YVGKTDSITISVWNHKKIHKKQGAGFLGCVRLLSNAISRLKDTGYQRLDLCKLNPSDTDA  120

2JQZ  119  VRGQIVVSLQSRD  131
           VRGQIVVSLQ+R
3PYC  121  VRGQIVVSLQTR   132
```

Fig. S5. Structural and sequence of Smurf1-C2 and Smurf2-C2. (A) Smurf1-C2 (magenta, PDB ID 3PYC, including 2 sulphates) and Smurf2-C2 (green, PDB ID 2JQZ) aligned. (**B)** Sequence alignment of Smurf1-C2 and Smurf2-C2, made using BLAST (blast.ncbi.nlm.nih.gov). Related to Table 2.

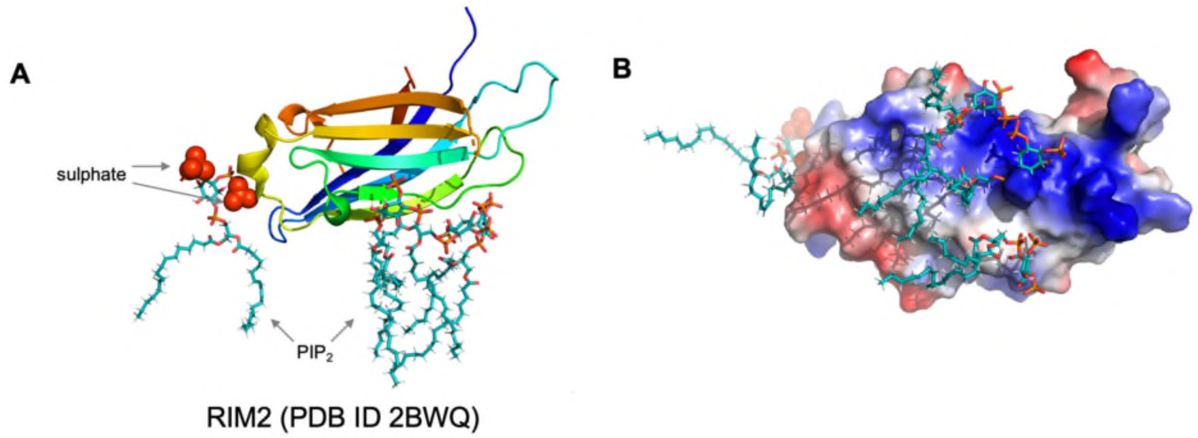

Fig. S6. Binding pocket of RIM2-C2. (A) Sulphates (in red/orange van der Waals format) in the crystal structure of RIM2 (PDB 2BWQ) are bound at the "bottom" of the C2 domain. The location of bound PIP_2 molecules (in 'bonds' format) from the atomistic simulation are also shown. (**B)** PIP_2 molecules observed bound in the simulation are shown alongside the surface of RIM2-C2 coloured according to its electrostatic potential (blue is positive charge and red is negative charge). Related to Table 2.

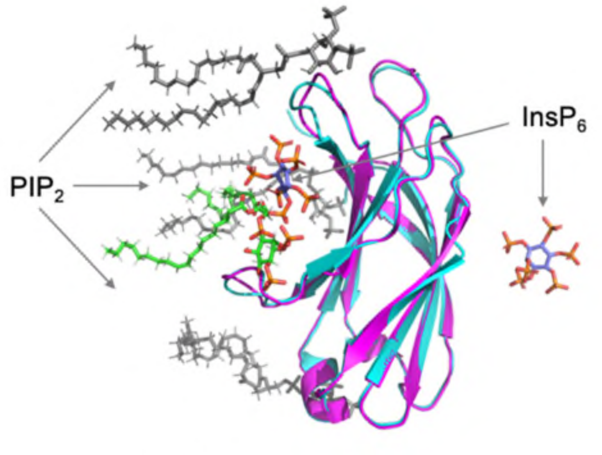

PI3KC2α (PDB ID 6BU0)

Fig. S7. Crystal structure and simulated PI3KC2α -C2. Crystal structure of PI3KC2α (PDB ID 6BU0; chain B; magenta) with 2 bound InsP$_6$ molecules, one at the front and one at the back. The simulated back-binding mode (cyan) structure is aligned with the crystal structure. One PIP$_2$ molecule (green) from the simulation was (in terms of headgroups) close to a bound InsP$_6$ molecule from the crystal (~0.85 nm) whereas the other PIP$_2$s (gray) bound at other locations. Related to Table 2.

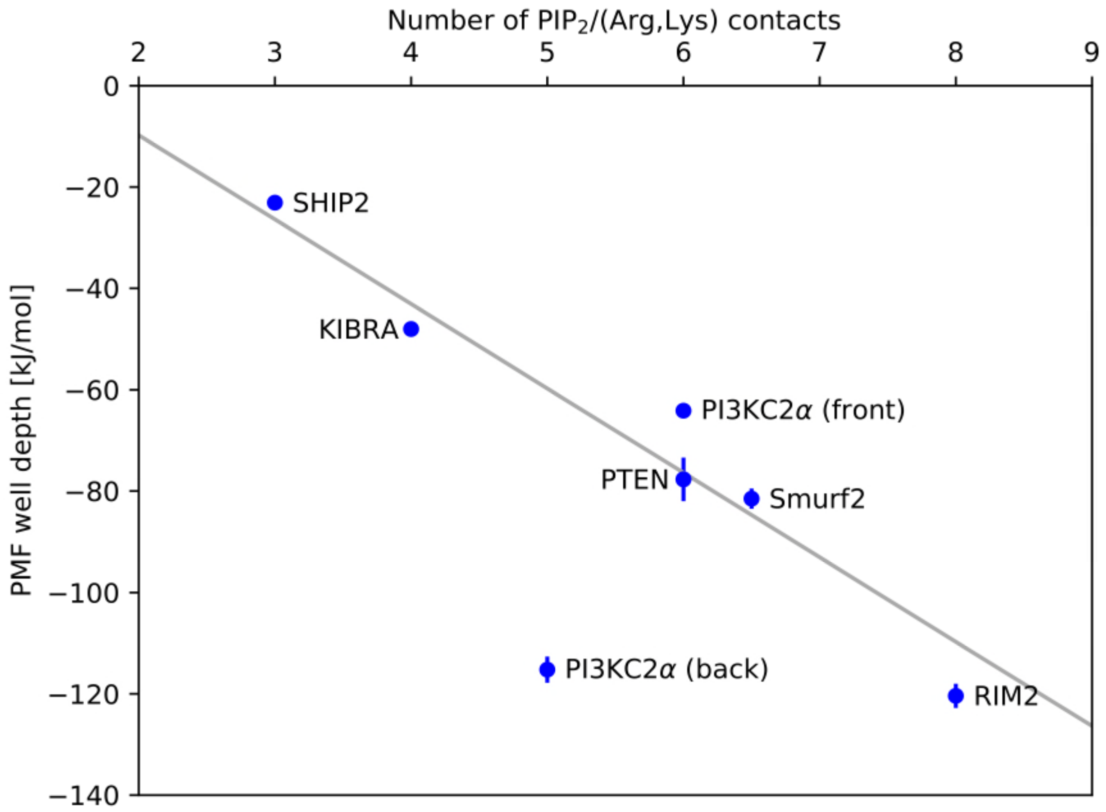

Fig. S8. PMF well depth correlates with the number of contacts between PIP_2 phosphates and (Arg, Lys) residues of C2. The binding poses from atomistic simulations reveal that the number of contacts between (Arg,Lys) sidechains with phosphates of PIP_2 are correlated with the depth of the free energy wells in the PMFs for membrane/C2 interactions, indicating that electrostatic interactions between basic sidechains of C2 and the PIP2 headgroups dominate but are not the sole determinants of the interactions. Related to Figure 5 and Table 1.
